# ACTIVE-GLU: Personalised modelling of physical activity-driven glucose dynamics in type 1 diabetes under free-living conditions

**DOI:** 10.1177/20552076261456506

**Published:** 2026-07-06

**Authors:** Ahmad Bilal, Hood Thabit, Paul W. Nutter, Simon Harper

**Affiliations:** 1 Department of Computer Science, 5292 The University of Manchester, Manchester, UK; 2 Diabetes, Endocrine and Metabolism Centre, Manchester Royal Infirmary, Manchester University NHS, Manchester, UK; 3 Division of Diabetes, Endocrinology and Gastroenterology, School of Medical Science, 5292 The University of Manchester, Manchester, UK

**Keywords:** type 1 diabetes, continuous glucose monitoring, physical activity, wearable devices, glucose dynamics, personalised modelling, rolling window, blood glucose prediction, free-living conditions, digital health

## Abstract

**Objective:**

Physical activity (PA) lowers blood glucose (BG) levels in individuals with Type 1 Diabetes Mellitus (T1DM), with effects varying by intensity and duration. Predicting BG fluctuations under free-living conditions, beyond structured exercise, helps reduce hypoglycaemia risk. This study aimed to model and predict BG responses across varying PA levels using personalised, activity-informed temporal patterns derived from continuous glucose monitoring (CGM) data.

**Methods:**

Fourteen adults with T1DM were monitored under free-living conditions using Garmin smartwatches for PA tracking and CGM devices for BG measurement. PA was quantified using step count and maxMotion intensity, and categorised into four levels: low, medium, high, and very high. A 10-day rolling window (moving average) was applied to each participant’s data to construct the personalised ACTIVE-GLU model, which estimates mean BG change at 15-minute intervals following PA.

**Results:**

The ACTIVE-GLU model accurately predicted BG changes across all PA intensities. The lowest average accuracy (81.67%) was observed under low PA, while the highest prediction error occurred during very high PA (MAE = 0.44 mmol/L, RMSE = 0.57 mmol/L, mean bound deviation = 0.38 mmol/L). Bland-Altman analysis confirmed close agreement between predicted and observed BG values, with negligible bias across intensity levels.

**Conclusion:**

In this exploratory cohort study, quantifying daily PA within a personalised temporal framework enables accurate prediction of BG responses in T1DM under real-world conditions. By incorporating PA intensity into BG dynamics, ACTIVE-GLU provides a personalised, PA-aware framework that may support interpretation of BG trends and inform future decision-support strategies. Future work will validate the model using virtual participants to simulate adaptive dosing interventions.

## Introduction

Hypoglycaemia induced by physical activity (PA) remains a significant concern for individuals with type 1 diabetes mellitus (T1DM), particularly during and after physical exertion.^1^ Hypoglycaemia is typically defined as a blood glucose (BG) level below 3.9 mmol/L, a threshold widely used in clinical guidelines to indicate clinically relevant low glucose,^2^ and may lead to symptoms such as dizziness, confusion, or loss of consciousness if severe. Increased PA promotes glucose uptake by muscle cells and enhances insulin sensitivity, which can lower BG levels and increase the risk of hypoglycaemia if not properly managed.^3^

Professional organisations and international consensus guidelines recommend maintaining BG levels within the range of 3.9-10 mmol/L to support optimal glycaemic control and reduce the risk of adverse events.^4,5^ Understanding how different PA intensities influence BG dynamics is therefore essential for developing effective management strategies in everyday settings. PA refers to any bodily movement produced by skeletal muscles that results in energy expenditure, including incidental activities such as walking, standing, or household tasks, while exercise represents a structured and planned subset of PA undertaken to improve or maintain physical fitness.^6^

In individuals with T1DM, both structured exercise and unstructured daily PA influence BG dynamics.^7,8^ However, while structured exercise has been extensively studied under controlled laboratory conditions, far less is known about BG responses to unstructured PA performed in free-living environments, which are inherently variable between individuals.^9,10^ Everyday activities, such as walking, climbing stairs, or household tasks, also influence BG responses. While low-intensity PA can improve insulin sensitivity with minimal immediate BG change,^11,12^ moderate PA often results in BG reductions during activity, and high-intensity PA may produce complex responses, including transient BG increases followed by delayed reductions.^9,13^ As these activities occur irregularly and interact with meals, insulin dosing, and other behavioural factors, predicting their impact on BG remains challenging in free-living environments.

In this study, we refer to everyday, irregular movements as non-standard PA. Unlike structured exercise, non-standard PA occurs spontaneously and varies widely in intensity and duration. We distinguish between standard PA, representing an individual’s typical daily activity level generally accommodated by routine insulin therapy, and non-standard PA, reflecting activity exceeding an individual’s usual daily average. Such increases may not be fully compensated by basal insulin dosing and can therefore lead to unexpected reductions in BG and increased risk of hypoglycaemia.^14^ As daily activity patterns vary between individuals, the threshold between standard and non-standard PA is inherently personalised.^15^

In our previous work, we conducted a temporal gradient analysis using continuous glucose monitoring (CGM) data alongside wearable-derived PA measurements to investigate whether increases in PA relative to an individual’s habitual baseline were associated with changes in BG trends. The findings demonstrated that non-standard increases in PA were frequently followed by steeper downward BG gradients, indicating that elevated activity levels can accelerate ongoing BG declines in free-living conditions.^15^ However, that study focused on identifying temporal associations rather than quantifying the magnitude of BG change attributable to PA. These results highlighted the importance of incorporating personalised activity patterns into predictive models of BG behaviour.

Recent advances in wearable technologies provide new opportunities to quantify PA in real-world settings. Wearable devices can continuously capture metrics such as step counts, heart rate, and activity intensity, enabling detailed monitoring of daily behaviour.^16,17^ Integrating such data into predictive models may support improved understanding of lifestyle factors influencing BG control and enable personalised recommendations for insulin or carbohydrate adjustments.^18^ Machine learning (ML) approaches have shown promise for predicting BG dynamics in structured exercise settings^19,20^; however, relatively few studies have addressed the complexity of spontaneous PA in daily life.^21^

Several algorithmic approaches have been proposed to predict exercise-induced hypoglycaemia using wearable and CGM data.^22^ Early predictive models combined CGM measurements with physiological signals such as heart rate to anticipate hypoglycaemic events during or after exercise.^23^ More recent approaches have integrated CGM data with contextual activity information to improve prediction accuracy during physical exertion.^22^ Other studies have relied solely on CGM-derived trends, using time-series analysis and pattern recognition to identify impending hypoglycaemia without external physiological inputs.^24^ While these approaches show promise, many rely on structured exercise protocols or fixed behavioural assumptions, limiting their applicability to spontaneous, free-living PA.

Predicting BG changes in free-living environments is particularly challenging because responses are influenced by multiple interacting variables, including insulin administration, meal timing, stress, and irregular activity patterns.^25,26^ Unplanned events, such as sudden increases in PA or unexpected meals, further increase variability and reduce prediction accuracy.^27^ Consequently, predictive models must adapt to individual behavioural patterns and physiological responses in dynamic real-world conditions.

To address these challenges, this paper presents ACTIVE-GLU, an adaptive precision framework designed to characterise personalised PA-BG relationships in individuals with T1DM. Unlike population-based approaches that rely on fixed thresholds, ACTIVE-GLU learns from each participant’s historical physiology and behavioural patterns using a rolling-window framework. By constructing personalised PA-BG profiles and iteratively incorporating new observations, the model continuously adapts to evolving routines and physiological states.

Using real-world data collected from wearable devices and CGM systems, the framework quantifies the relationship between varying PA intensities and subsequent BG changes at 15-minute intervals. Unlike many publicly available CGM datasets,^28,29^ which often lack detailed annotation of PA timing, this study incorporates wearable-derived PA data aligned with glucose measurements under free-living conditions, enhancing ecological validity. Model performance is evaluated using prediction-interval coverage and error metrics under different PA conditions. The long-term aim is to support adaptive decision-making for insulin dosing and carbohydrate intake, with potential future integration into bolus calculators.^30^ Such systems may help individuals with T1DM manage BG fluctuations more effectively in free-living conditions.

## Methodology

We develop a predictive model for BG levels based on varying intensities of PA, categorised as low, medium, high, and very high. We implemented a minimum of a 10-day rolling window for each individual to create personalised models. For each individual, the model predicts the average change in BG levels 15 minutes after different PA levels. This is achieved by calculating the mean and standard deviation (STD) of BG changes across a rolling window of at least 10 and up to 20 days of that participant’s data.

### Participants

The analysis utilised data from a cohort of 14 individuals with T1DM who participated in our free-living study.^31^ Of these, 6 participants were confirmed to be using closed-loop insulin delivery systems (Tandem t:slim X2, MiniMed 780G, or Omnipod 5), while the remainder used either multiple daily injections (MDI) or non-automated insulin pumps. Participants adjusted their insulin based on carbohydrate intake, BG levels, and PA. Table 1 provides demographic and device-related details.

**Table 1.** Summary of the 14 participants included in the ACTIVE-GLU modelling cohort. Data were collected over 12 weeks under free-living conditions, integrating continuous glucose monitoring (CGM) and activity. CGM manufacturers: D = Dexcom (Clarity), L = Libre (LibreView), M = Medtronic (CareLink). “D to M” indicates a participant who transitioned from Dexcom to Medtronic during the study. Gender: 0 = male, 1 = female. Insulin therapy: MDI = multiple daily injections; Pump = insulin pump operated in open-loop mode (Tandem t:slim X2, MiniMed 780G, or Omnipod 5). One pump user (ID2304) transitioned to a closed-loop configuration partway through the study. HbA1c values are expressed in mmol/mol and correspond to the most recent clinically measured value prior to study enrolment. N/A = not available.

Participant ID	CGM manufacturer (D/L/M)	Start date	End date	Gender (0=Male, 1=Female)	Age (years)	Weight (kg)	Height (m)	Insulin therapy (Pump/MDI)	HbA1c (mmol/mol)
ID2301	D	6-Oct-2023	29-Dec-2023	1	25	64.0	1.62	Pump	51
ID2302	L	1-Oct-2023	24-Dec-2023	1	29	61.0	1.74	MDI	43
ID2304	D to M	17-Oct-2023	5-Feb-2024	1	29	82.0	1.65	Pump	N/A
ID2306	L	18-Oct-2023	10-Jan-2024	1	50	63.0	1.64	MDI	41
ID2308	M	4-Dec-2023	26-Feb-2024	0	59	70.0	1.77	Pump	39
ID2309	M	5-Feb-2024	1-May-2024	1	59	103.0	1.68	Pump	N/A
ID2310	M	13-Oct-2023	17-Jan-2024	0	70	88.0	1.85	Pump	51
ID2313	L	10-Nov-2023	2-Feb-2024	0	39	127.0	1.82	MDI	51
ID2314	L	6-Nov-2023	29-Jan-2024	0	61	86.0	1.85	MDI	51
ID2320	D	1-Dec-2023	23-Feb-2024	1	46	58.2	1.65	Pump	50
ID2401	L	19-Jan-2024	1-May-2024	0	46	85.0	1.82	MDI	N/A
ID2403	L	12-Mar-2024	25-Jun-2024	0	23	70.0	1.75	MDI	58
ID2404	L	11-Mar-2024	10-Jun-2024	1	37	76.0	1.60	MDI	53
ID2405	L	3-Jun-2024	3-Sep-2024	0	52	85.7	1.85	MDI	54

Participants were aged 18-70 years, had T1DM for more than 2 years, had no confounding health conditions, and engaged in regular daily activities in a free-living environment. Although this age range spans younger and older adults, potentially introducing variability in PA levels and physiological responses, the modelling approach is inherently personalised. Predictions are derived from each participant’s own historical data using a personalised rolling window, enabling the model to adapt to person-specific behavioural and physiological patterns rather than relying on population-level assumptions. This design mitigates the impact of inter-individual variability, including age-related differences in PA patterns and BG regulation.

### Materials

#### Glucose monitoring

Participants utilised Dexcom G6, Freestyle Libre 2, and Medtronic Guardian 4 CGM devices to monitor their BG levels (mmol/L), which they were already using as part of their routine clinical management. Data were collected at 5 to 15-minute intervals, depending on the device, throughout the study. For consistency with previous literature, the term BG is used throughout this paper to denote interstitial glucose concentrations derived from CGM sensors. Although CGM readings are obtained from subcutaneous tissue rather than capillary blood, they closely approximate BG dynamics and are routinely used in BG prediction and modelling studies.^32^

To ensure consistency across devices with different sampling frequencies and formats, a standardised preprocessing and quality control pipeline was applied, as described in the original dataset paper.^31^ Raw CGM data were cleaned by removing missing, duplicate, and invalid entries, retaining only time-stamped sensor BG values. As devices recorded data at varying intervals (5–15 minutes), timestamps were normalised by rounding to the nearest 5-minute mark.

#### Activity monitoring

PA was assessed using the Garmin Forerunner 45, a wrist-worn wearable smartwatch designed for continuous activity tracking.^33^ The device incorporates embedded accelerometer sensors to capture movement-based data, which are processed using proprietary algorithms to estimate PA metrics. Specifically, it measures “Step Count” and “Maximum Motion Intensity”, the latter reflecting peak acceleration within each 15-minute epoch and serving as an indicator of movement intensity. These metrics are widely used to quantify activity levels and to distinguish between sedentary and active states, including sleep and wake intervals.^34^

### Data collection

All participants were encouraged to maintain their usual lifestyle, without altering their dietary habits or PA. This approach ensured that the data collected accurately reflected their everyday behaviours, enabling a clearer understanding of their usual routines without the influence of external factors. We selected participant data spanning up to 12 weeks for consistency and relevance. Haemoglobin A1C (HbA1c) reflects average BG control over approximately 8-12 weeks,^35^ allowing our analysis to align with long-term glycemic trends and lifestyle factors.

#### Participants recruitment

Participants were recruited through a combination of clinical and community-based approaches in Manchester, United Kingdom. Clinical recruitment took place through the diabetes outpatient services at Manchester Royal Infirmary (MRI), part of Manchester University NHS Foundation Trust. Additional participants were recruited through advertisements (posters and flyers) placed across the University of Manchester campus and surrounding facilities, as well as through online outreach, consistent with the dataset protocol.^31^

#### BG and PA data collection

Participants attended dedicated healthcare provider clinics, where they provided informed consent for data sharing and analysis for this project. For participants unable to attend in person, consent and onboarding were conducted via secure online meetings. BG reports were gathered from various platforms to ensure a smooth data collection process, including Dexcom Clarity,^36^ LibreView,^37^ and Medtronic CareLink,^38^ and were collected during routine clinical visits.

PA data were collected using the Garmin Forerunner 45 smartwatch. A custom API was developed for this research^39^ to seamlessly integrate with the Garmin Connect Developer Program, allowing retrieval of real-time, historical, and batch data. The API captured detailed raw activity data, including HEALTH-Epochs,^40^ providing structured time-series datasets. Developed using a Python framework, the API was deployed on a secure server at The University of Manchester.^41^ Participants provided consent through the Garmin API Developer Platform to share their data for collection and analysis. This study was conducted and reported in accordance with the Strengthening the Reporting of Observational Studies in Epidemiology (STROBE) guidelines.^42^ The completed checklist is provided in Supplementary Information section.

#### Data selection

Although insulin and carbohydrate intake are central to glycaemic control, these variables were deliberately excluded from the current modelling phase due to their inconsistent availability and limited reliability in free-living datasets. In real-world conditions, meal and bolus records are often incomplete, inaccurately timestamped, or imprecise in carbohydrate estimation, while insulin logs may omit manual boluses or reflect delayed corrections. Such inconsistencies introduce substantial measurement error and temporal misalignment with CGM and PA data, reducing interpretability and predictive validity. Including dietary and insulin data would also obscure the independent effect of PA, as compensatory carbohydrate intake or insulin adjustments can weaken its observed glycaemic impact. ACTIVE-GLU therefore isolates the PA–BG relationship using a rolling-window framework.

CGM and wearable-derived PA data, by contrast, are objective, time-stamped, and automatically recorded, providing a reliable basis for modelling. By focusing on PA alone, we aim to characterise its specific contribution to BG fluctuations, which is often difficult to isolate when analysed alongside insulin dosing and meal intake. Establishing this PA-aware baseline allows future extensions of the framework to incorporate insulin and carbohydrate data once these inputs can be synchronised automatically from closed-loop devices or validated logging platforms. As several participants in this study used open-loop or manual insulin systems (MDI or non-automated pumps), the predictive logic of ACTIVE-GLU was primarily developed and evaluated under behaviourally controlled dosing conditions, providing clinically relevant insights for individuals who self-manage insulin delivery.

#### Data aggregation and classification

Two key metrics were prioritised: *step count* and *maxMotion*. The composite PA score was derived by multiplying these values per epoch, producing a continuous measure reflecting both movement volume and exertion intensity (see Supplementary Algorithm 1). Raw PA data were classified by Garmin into Walking, Running, Generic, and Sedentary states; aggregated values per 15-minute epoch were then assigned to one of four personalised quantile-based intensity levels (low, medium, high, very high), as formalised in Equation (10) and detailed in Supplementary Algorithm 2. PA occurring below each participant’s mean daily level was classified as standard PA; activity exceeding this threshold was classified as non-standard PA and formed the basis for subsequent modelling.^7,14^

#### Data merging

PA and BG datasets were aligned to uniform 15-minute epochs and merged on timestamp; intervals with missing values in either signal were excluded.^43,44^ Data were restricted to waking hours (06:00-midnight). Only intervals showing a BG decrease (Δ*BG*(*t*) > 0) were retained, isolating PA-induced hypoglycaemic risk as the primary prediction target. This filtering was applied causally before any rolling-window construction, avoiding look-ahead bias. Full procedural details are provided in Supplementary Algorithms 3-4.

### Data analysis

To aid interpretation and explicitly illustrate how wearable-derived PA data (from the Garmin Forerunner 45) are integrated with CGM data within the ACTIVE-GLU framework, we present the overall algorithmic workflow in Table 2. This outlines the sequential steps from PA processing to BG drop prediction using a 10-day rolling window, supporting both technical clarity and reproducibility across clinical and data science domains. A rolling window (moving average window) is a widely used technique in time series analysis and forecasting that smooths data by averaging sequential observations over a specified window size.^45,46^ This approach helps identify trends by reducing data noise, making it well-suited for predictive modelling.

**Table 2.** Structured pseudocode summarising the ACTIVE-GLU analytical workflow. The table outlines the main modelling pipeline, including wearable-derived PA preprocessing, participant-specific PA quantile classification, CGM preprocessing, PA–BG data merging, and rolling-window prediction. Detailed procedural implementations corresponding to each step are provided in Supplementary Algorithms 1–5.

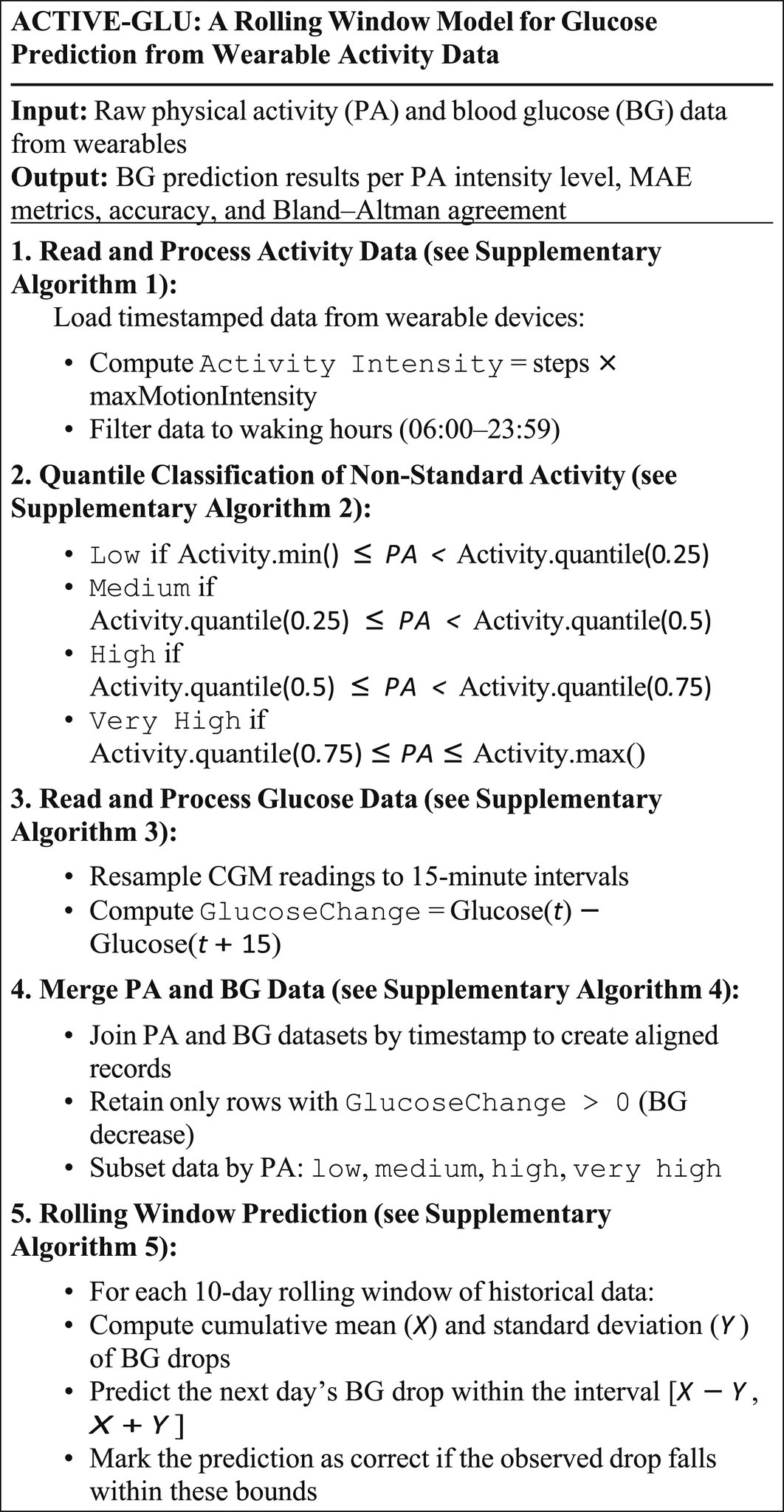

Traditional moving average calculations, while effective for smoothing temporal signals, often lack adaptability to the unique dynamics of diverse datasets, limiting their performance in complex scenarios. Recent research has therefore focused on enhancing the flexibility of moving-average approaches within rolling-window frameworks. For instance,^47^ proposed the Self-Attentive Moving Average, which integrates a self-attention mechanism to assign dynamic weights to time steps based on their relevance, improving traditional methods. Another study^45^ introduced an adaptive moving-average approach that dynamically adjusts weights to accommodate variations across datasets, demonstrating its effectiveness across diverse time-series applications. These innovations highlight the potential of combining classical moving-average calculations with modern computational advances to improve their accuracy and applicability for predictive tasks.

We adopted a similar approach,^45,47^ which uses a rolling-average window to predict BG changes at different levels of PA. After combining various levels of PA with BG data, we calculated changes in BG levels for consecutive 15-minute intervals for each participant to develop a personalised model. For instance, if a participant had a BG value of 6.7 *mmol*/*L* at 10:00 and 5.3 *mmol*/*L* at 10:15 for a specific PA level (low, medium, high, or very high), the change in BG would be 1.4 mmol/L. Once we established a BG change for all participants, we created a rolling window model using daily data spanning at least 10 days to predict the average drop and STD in BG changes for each PA level, where we had cumulative data for more than 10 days. The complete algorithm is presented in the Supplementary Information in Algorithm 5.

To predict the average changes in BG for the next unseen day, we applied a rolling window of size *n* over the preceding *n* days of PA and BG data. For each iteration, the model used the most recent *n* days (minimum 10) to compute the mean and STD of 15-minute BG changes after PA, then generated a prediction for the following day. After each prediction, the window advanced by one day, discarding the oldest record and including the newest, ensuring that each forecast was based exclusively on historical data preceding the prediction target. This rolling structure allows ACTIVE-GLU to continuously update and generalise from past behaviour to forecast unseen daily responses. When a participant’s behaviour changes, such as altered carbohydrate intake, insulin dosing, or PA routine, the model temporarily reflects this as increased variability (destabilised STD). However, once the new pattern persists for several days, the STD re-stabilises and the model re-adapts, maintaining predictive reliability while naturally tracking evolving physiological and behavioural dynamics.

For each PA level, we computed the mean BG drop during the window and used this to estimate the predicted BG change for the following day. However, since BG responses to PA can vary, relying solely on the mean does not capture the full range of possible outcomes. Therefore, we also calculated the STD to establish upper and lower bounds around each mean, thereby generating more realistic predictive intervals for the BG drop the next day. Calculating STD is required for quantifying the spread of data points relative to the mean.^48^ It provides a direct and interpretable measure of variability in BG responses and PA levels, enabling more clinically meaningful analysis. Additionally, we had over two weeks of data to provide a basis for understanding long-term trends in PA effects on BG. As shown in study,^49^ a CGM sampling duration of approximately 10–14 days may provide reasonable correlation with long-term glycaemic metrics, thereby enhancing the model’s predictive accuracy and applicability.

**Figure 1.**
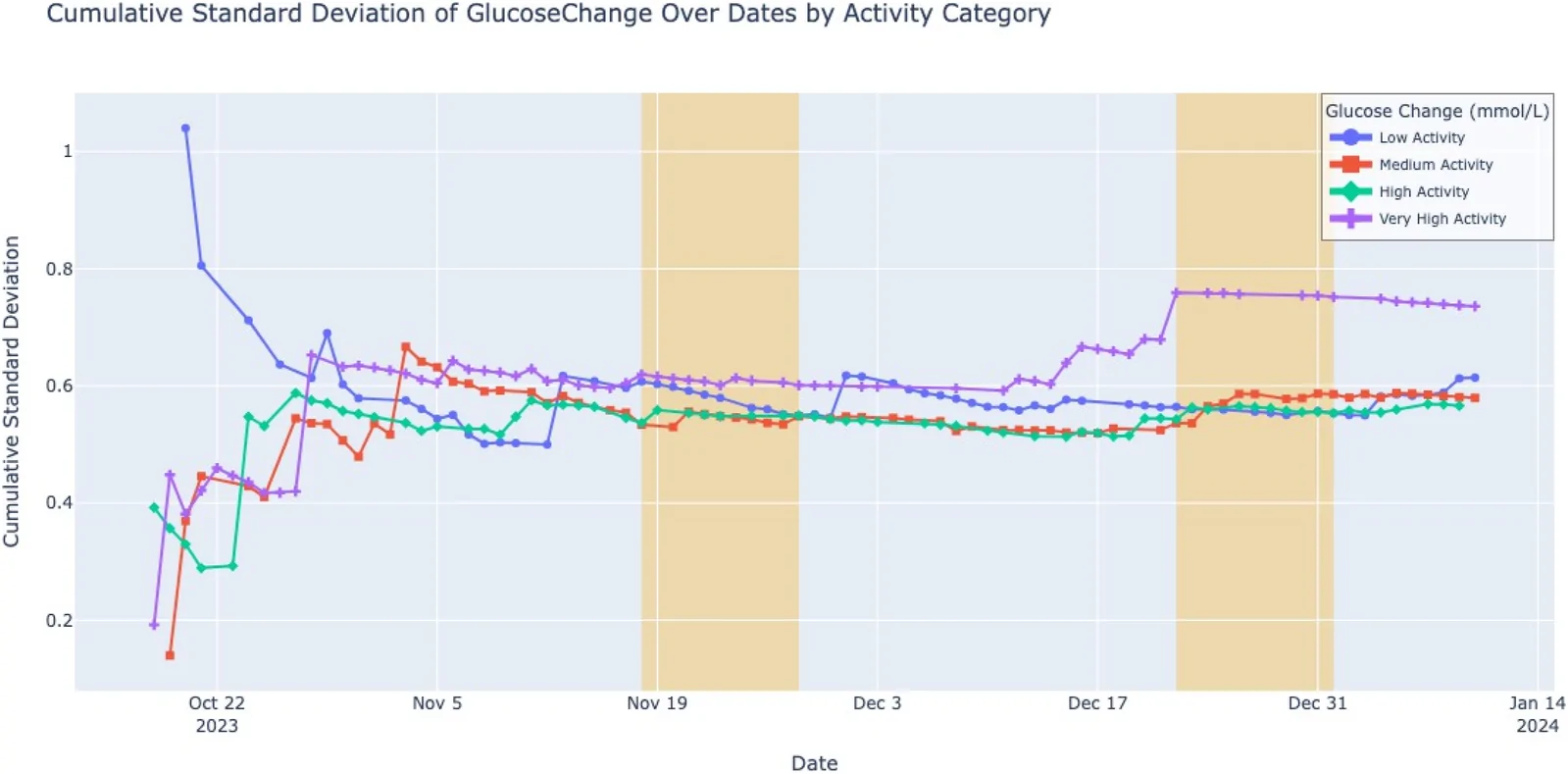
Temporal interplay: A standard deviation (STD) analysis illustrating the cumulative STD of glucose changes across physical activity (PA) levels over time for a representative individual participant (ID2306) from the study cohort (total cohort *n* = 14). Glucose changes (Δ*BG*) are expressed in mmol/L and derived from CGM measurements recorded at 15-minute intervals and aggregated daily. Two shaded regions highlight periods where the STD begins to stabilise, suggesting consistent variability in BG response under similar activity patterns. These stable zones may reflect physiological adaptation or routine behavioural patterns. Activity was represented by a composite activity score calculated as the product of step count and Garmin’s maxMotion intensity.

The analysis revealed that, for most participants, the STD in BG fluctuations tended to stabilise after a minimum of 8–10 days, and for a few participants, around 15-20 days, as shown in Figure 1. This observation informed our decision to implement a minimum of a 10-day rolling window for data processing across participants. Stabilisation of STD in BG changes suggests that the body has reached an adaptive state, allowing us to derive reliable results on the decline in BG fluctuations across different levels of PA. Additionally, this stabilisation reflects consistent variability across observations or subsets, underscoring the importance of STD as a critical metric in data analysis.^50^

While STD may increase or decrease at later stages due to changes in PA intensity or lifestyle, we anticipate the STD stabilising again with another minimum stretch of 8–10 consecutive days. This expectation aligns with the patterns observed earlier in the study, where consistent PA over such periods led to stabilisation, supporting the use of rolling windows to adapt to new physiological states.

We utilised the data from the first 10 days to build the personalised model using a rolling window approach, calculating the average drop in BG using Equation (1) and the STD using Equation (2). By calculating the mean as outlined in Equation (1), we determined the average drop in BG for each level of PA. For instance, if an individual engages in a medium level of PA, they will experience an average drop in BG after 15 minutes of *Xmmol*/*L* and a STD of *Y*. This *X* ± *Y* mmol/L estimate provides an indication of the expected BG change associated with a given PA level and may support interpretation of glucose trends in relation to PA. The complete methodology for developing this model is detailed in Algorithm 5 in the Supplementary Information.

(1) $$\begin{array}{cc} X & =\text{mean}\left(\text{cumulative\_data}\right)\\ & \in \text{daily mean calculated as:}\\ & \text{Day }1,\;\left(\text{Day }1\;+\text{ Day }2\right),\;\left(\text{Day }1\;+\text{ Day }2\;+\text{ Day }3\right),\\ & \ldots ,\;\left(\text{Day }1\;+\text{ Day }2\;+\text{ Day }3\ldots +\text{Last Day}\right). \end{array}$$

(2) $$\begin{array}{cc} Y & =\text{STD}\left(\text{cumulative\_data}\right)\\ & \in \text{daily STD calculated as:}\\ & \text{Day }1,\;\left(\text{Day }1\;+\text{ Day }2\right),\;\left(\text{Day }1\;+\text{ Day }2\;+\text{ Day }3\right),\\ & \ldots ,\;\left(\text{Day }1\;+\text{ Day }2\;+\text{ Day }3\ldots +\text{Last Day}\right). \end{array}$$

**Figure 2.**
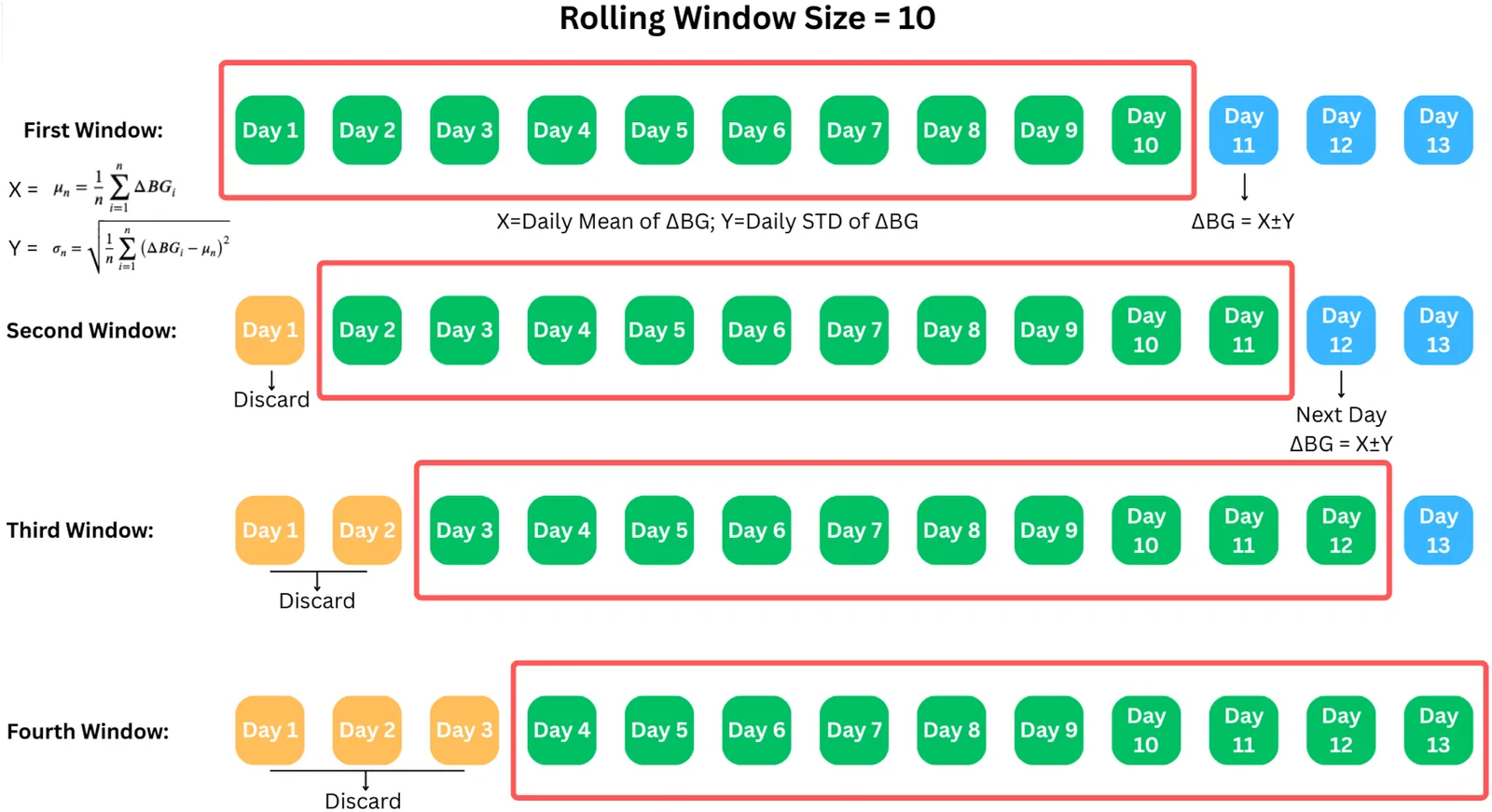
Illustration of the 10-day rolling window framework used for predicting glucose drops (Δ*BG*, mmol/L) from wearable-derived activity data. Each window (red box) spans 10 consecutive days of observations per participant and advances by 1 day at each iteration. For every window, the cumulative mean (*μ*_*n*_) and standard deviation (*σ*_*n*_) of daily glucose drops are computed and used to determine whether the following day’s glucose change falls within the predicted interval [*μ*_*n*_ ± *σ*_*n*_]. Mean/STD = (Day 1 (ΔBG)), (Day 1 (ΔBG) + Day 2 (ΔBG)), …, (Day 1 (ΔBG) + Day 2 (ΔBG) + …+ Day 10 (ΔBG)).

A rolling window approach Figure 2 was applied to sequential 10-day intervals of merged data of PA and BG. For each window, the model computed the cumulative mean as calculated in Equation (3) and STD of daily BG changesEquation 4, which were then used to predict whether the next day’s BG drop would fall within the range [*μ*_*n*_ ± *σ*_*n*_]. A prediction window was considered correct if the observed BG change (Δ*BG*) lay within the predicted interval ([*μ*_*n*_ ± *σ*_*n*_]).

(3) $$\mu_{n}=\frac{1}{n}\overset{n}{\underset{i=1}{\sum }}\Delta BG_{i}$$

(4) $$\sigma_{n}=\sqrt{\frac{1}{n}\overset{n}{\underset{i=1}{\sum }}{\left(\Delta BG_{i}-\mu_{n}\right)}^{2}}$$

with ACTIVE-GLU now developed, we can estimate the expected BG change over the next 15 minutes for each PA level: low, medium, high, and very high. These predictions are derived from Equation (1) and Equation (2). The Results section presents these analyses, showing the average BG change every 15 minutes across PA levels.

## Results

We focus on addressing PA that is not compensated for by basal insulin, particularly PA that exceeds an individual’s average daily PA level. For participants using MDI or non-automated pump systems, this can inform decisions around basal insulin or carbohydrate intake adjustments. However, only adjustments to carbohydrate intake are typically possible in closed-loop pump users, since the system’s algorithm automatically regulates basal insulin. Moving forward, our prediction algorithm, developed using data from 14 participants (including 6 on closed-loop systems), assumes that BG responses can be predicted when at least 10 consecutive days of PA and BG data are available. Once the STD in BG changes stabilises, we estimate that within a 15-minute window, BG levels can be represented as described in Equation (5), where *X* denotes the average change in BG and *Y* its STD.

(5) Change in BG=X±Y

To evaluate model performance across the four PA categories (*Low*, *Medium*, *High*, and *Very High*), we employed standard error and agreement metrics commonly used in BG prediction literature. These include accuracy, mean absolute error (MAE), root mean squared error (RMSE), and mean bound deviation. Accuracy quantifies the proportion of correctly predicted next-day BG changes within the interval defined by the predicted mean ±STD, while MAE and RMSE measure absolute and squared deviations between predicted and observed changes, respectively. Mean bound deviation captures the average magnitude by which predictions fall outside their expected range, providing additional clinical interpretability.

Accuracy was calculated as:

(6) $$\text{Accuracy}=\frac{\text{Correct Windows}}{\text{Total Windows}}\times 100,$$

where a prediction is considered correct if the actual BG change (Δ*BG*) lies within the predicted interval ${\hat{\Delta BG}}_{\text{mean}}\pm \sigma_{\hat{\Delta BG}}$ . A prediction window was defined as correct window if the observed BG change (Δ*BG*) fell within the predicted interval defined by the predicted mean BG change and its associated variability, expressed as ${\hat{\Delta BG}}_{\text{mean}}\pm \sigma_{\hat{\Delta BG}}$ . This interval represents the expected range of BG responses derived from the rolling-window model. If the observed BG change occurred within this range, the window was classified as a correct prediction; otherwise, it was considered incorrect. The predicted versus actual mean BG changes are visualised using scatter plots with overlaid error bars, representing the uncertainty range (predicted STD) for each participant and PA level. These visualisations, together with participant-level bar charts and Bland-Altman analyses, provide an assessment of model performance across PA levels.

We consider MAE < 1 mmol/L indicative of acceptable predictive accuracy, as this magnitude of error is comparable to the inherent variability observed in CGM measurements and remains small relative to typical BG action ranges used in clinical decision-making.^51^ The proposed framework does not rely solely on point predictions; instead, predictions are interpreted together with their associated variability, expressed as a range of expected BG responses (*X* ± *Y*). This range-based interpretation enables users to anticipate potential BG declines during PA and take preventive actions, such as consuming additional carbohydrates before activity or adjusting bolus insulin, particularly when the predicted lower bound approaches clinically relevant thresholds such as hypoglycaemia (3.9 mmol/L).

The results presented in Table 3 (Low PA), Table 4 (Medium PA), Table 5 (High PA), and Table 6 (Very High PA) provide a summary of the model’s performance across different PA intensities are discussed in details in the Result Analysis Section. Each table corresponds to a specific PA level and gives participant-level prediction metrics based on a rolling-window analysis of BG trends. Overall, the model showed high prediction performance across all PA levels, with higher accuracy and lower error metrics in the low and medium PA categories. Although error metrics increased at very high PA levels, for some participants, most predictions remained within clinically acceptable bounds, underscoring the model’s potential utility for real-world BG forecasting.

**Table 3.** Participant-wise prediction performance under the Low PA level. Accuracy remained high across most individuals, with minimal deviation from predicted bounds. Participants ID2301 and ID2304 achieved 90%+ accuracy, indicating reliable alignment between predicted and actual BG trends.

Participant ID	Accuracy (%)	Predicted BG Change - Average (mmol/L)	Predicted BG Change - std. Dev. (mmol/L)	Actual next Day Mean (mmol/L)	lMAE (mmol/L)	RMSE (mmol/L)	Mean Bound Deviation (mmol/L)	Correct/Total Windows
ID2301	91.43	0.74	0.72	0.64	0.42	0.52	0.49	32/35
ID2302	80.85	0.55	0.51	0.59	0.36	0.53	0.37	76/94
ID2304	91.67	0.66	0.56	0.78	0.36	0.68	1.47	22/24
ID2306	81.82	0.64	0.57	0.71	0.35	0.44	0.26	45/55
ID2308	75.00	0.61	0.49	0.58	0.36	0.45	0.26	12/16
ID2309	86.36	0.66	0.62	0.71	0.36	0.52	0.57	38/44
ID2310	78.72	0.73	0.63	0.71	0.45	0.64	0.44	37/47
ID2313	85.29	0.71	0.61	0.71	0.38	0.47	0.33	58/68
ID2314	83.02	0.72	0.54	0.76	0.34	0.48	0.33	44/53
ID2320	69.09	0.42	0.31	0.38	0.24	0.31	0.13	38/55
ID2401	72.50	0.66	0.60	0.70	0.41	0.51	0.22	29/40
ID2403	88.24	0.67	0.66	0.58	0.35	0.47	0.29	30/34
ID2404	86.67	0.70	0.52	0.63	0.31	0.38	0.17	39/45
ID2405	78.00	0.72	0.56	0.71	0.35	0.42	0.15	39/50

**Table 4.** Prediction summary for participants during the Medium PA category. Accuracy remains high again, though some participants (again ID2304) exhibit lower prediction reliability. Despite this, errors remain within acceptable mean bounds.

Participant ID	Accuracy (%)	Predicted BG Change - Average (mmol/L)	Predicted BG Change - std. Dev. (mmol/L)	Actual next Day Mean (mmol/L)	MAE (mmol/L)	RMSE (mmol/L)	Mean Bound Deviation (mmol/L)	Correct/Total Windows
ID2301	86.67	0.61	0.45	0.63	0.29	0.40	0.36	26/30
ID2302	88.04	0.63	0.56	0.65	0.33	0.46	0.44	81/92
ID2304	65.22	0.57	0.42	0.68	0.34	0.51	0.33	15/23
ID2306	83.33	0.71	0.58	0.78	0.39	0.53	0.44	50/60
ID2308	72.22	0.49	0.35	0.52	0.24	0.31	0.17	13/18
ID2309	80.00	0.68	0.62	0.75	0.44	0.66	0.60	32/40
ID2310	87.50	0.77	0.72	0.74	0.42	0.49	0.18	42/48
ID2313	81.43	0.73	0.62	0.70	0.37	0.46	0.17	57/70
ID2314	89.06	0.88	0.71	0.84	0.37	0.56	0.51	57/64
ID2320	82.61	0.55	0.47	0.53	0.32	0.42	0.22	38/46
ID2401	87.80	0.63	0.60	0.65	0.38	0.43	0.20	36/41
ID2403	84.78	0.55	0.46	0.52	0.25	0.34	0.29	39/46
ID2404	85.37	0.72	0.63	0.71	0.38	0.51	0.40	35/41
ID2405	82.35	0.77	0.63	0.73	0.36	0.45	0.21	42/51

**Table 5.** Detailed evaluation for the High PA category. While average accuracy is high, several participants show increased RMSE and bound deviation, indicating greater BG instability. The model continues to perform within acceptable mean bound thresholds, again showing reliable alignment between predicted and actual BG trends.

Participant ID	Accuracy (%)	Predicted BG Change - Average (mmol/L)	Predicted BG Change - std. Dev. (mmol/L)	Actual next Day Mean (mmol/L)	MAE (mmol/L)	RMSE (mmol/L)	Mean Bound Deviation (mmol/L)	Correct/Total Windows
ID2301	96.43	0.78	0.66	0.77	0.32	0.51	1.52	27/28
ID2302	81.25	0.60	0.55	0.67	0.36	0.57	0.51	78/96
ID2304	76.19	0.77	0.71	0.90	0.43	0.67	0.53	16/21
ID2306	78.95	0.70	0.55	0.73	0.36	0.46	0.25	45/57
ID2308	83.33	0.58	0.49	0.69	0.44	0.83	1.09	15/18
ID2309	79.07	0.80	0.71	0.77	0.50	0.59	0.29	34/43
ID2310	77.78	0.69	0.59	0.65	0.38	0.47	0.24	28/36
ID2313	80.00	0.85	0.71	0.80	0.48	0.64	0.43	48/60
ID2314	77.78	0.96	0.65	0.97	0.43	0.51	0.22	49/63
ID2320	80.39	0.62	0.42	0.56	0.31	0.39	0.27	41/51
ID2401	84.38	0.56	0.52	0.57	0.33	0.41	0.29	27/32
ID2403	81.58	0.69	0.61	0.74	0.45	0.68	0.68	31/38
ID2404	79.55	0.85	0.59	0.83	0.43	0.49	0.13	35/44
ID2405	94.55	0.72	0.62	0.66	0.28	0.34	0.26	52/55

**Table 6.** Prediction summary for the Very High PA category. Despite the increased intensity, the model maintained a mean accuracy of ≈ 82%. A few participants (ID2308) experienced higher variability but achieved high accuracy. The overall predictions remained in the predicted interval.

Participant ID	Accuracy (%)	Predicted BG Change - Average (mmol/L)	Predicted BG Change - std. Dev. (mmol/L)	Actual next Day Mean (mmol/L)	MAE (mmol/L)	RMSE (mmol/L)	Mean Bound Deviation (mmol/L)	Correct/Total Windows
ID2301	83.78	0.89	0.67	0.84	0.43	0.51	0.19	31/37
ID2302	77.65	0.67	0.53	0.67	0.34	0.45	0.26	66/85
ID2304	76.92	0.79	0.62	0.89	0.43	0.56	0.34	20/26
ID2306	86.21	0.85	0.72	0.76	0.48	0.61	0.46	50/58
ID2308	90.00	1.29	1.34	1.28	0.88	1.20	1.89	9/10
ID2309	87.18	0.65	0.65	0.65	0.44	0.56	0.60	34/39
ID2310	88.57	0.86	0.68	0.83	0.38	0.48	0.30	31/35
ID2313	79.37	0.77	0.67	0.70	0.39	0.52	0.27	50/63
ID2314	76.92	1.34	0.81	1.18	0.49	0.61	0.25	40/52
ID2320	80.77	1.05	0.70	1.06	0.46	0.56	0.29	42/52
ID2401	82.86	0.67	0.61	0.77	0.41	0.61	0.49	29/35
ID2403	80.00	0.86	0.72	0.86	0.45	0.56	0.25	32/40
ID2404	84.78	0.89	0.68	0.90	0.42	0.54	0.34	39/46
ID2405	85.19	1.01	0.96	0.95	0.52	0.72	0.59	46/54

### Results analysis

We assessed prediction accuracy by determining whether the observed BG change fell within the range defined by the predicted mean ± predicted STD, which captures expected variability around the prediction. For instance, if the model predicted a BG change of 0.70 mmol/L ± 0.10 mmol/L, any observed BG change between 0.60 mmol/L and 0.80 mmol/L would be considered a correct prediction. A prediction was considered correct if the observed BG change satisfied the condition defined in Equation (7):

(7) $${\hat{\Delta BG}}_{\text{mean}}-\sigma_{\hat{\Delta BG}}\leq \Delta BG\leq {\hat{\Delta BG}}_{\text{mean}}+\sigma_{\hat{\Delta BG}}$$

where ${\hat{\Delta BG}}_{\text{mean}}$ denotes the predicted mean BG change, Δ*BG* represents the observed BG change, and $\sigma_{\hat{\Delta BG}}$ indicates the predicted standard deviation.

#### Accuracy (prediction-interval coverage)

Accuracy measures how frequently the BG change falls within the predicted range (Predicted BG change ± Predicted STD). This metric quantifies the model’s ability to accurately predict BG fluctuations and estimate the associated physiological variability induced by different PA intensities. In Table 3 (Low PA), the lowest accuracy was observed for participant ID2320, who had a mean bound deviation of 0.13 mmol/L. This relatively small deviation suggests that although the model occasionally misclassified the trend, the actual BG changes remained within the predicted bounds, implying a minor clinical impact. In contrast, across Tables (4, 5 and 6), covering medium, high and very high PA levels, the lowest accuracies were consistently observed for ID2304, with mean bound deviations of 0.33 mmol/L, 0.53 mmol/L and 0.34 mmol/L, respectively. This may reflect increased physiological variability in ID2304’s BG responses to higher levels of PA, potentially due to inconsistent recovery patterns, varying insulin sensitivity, or factors such as stress, meal timing, or insulin delivery.

While the Tables (3, 4, 5, 6) provide participant-specific prediction metrics, we also computed generalised mean accuracy for each PA intensity level to assess overall model performance. These aggregated figures reflect the model’s ability to generalise across individuals within each PA category. The calculated accuracies and correct prediction counts for each PA level are as follows:

• **Low PA:** 81.67% accuracy (539 correct predictions out of 660 total prediction windows).
• **Medium PA:** 84.03% accuracy (563 correct predictions out of 670 total prediction windows).
• **High PA:** 81.93% accuracy (526 correct predictions out of 642 total prediction windows).
• **Very High PA:** 82.12% accuracy (519 correct predictions out of 632 total prediction windows).

The accuracy across PA intensities shows that the rolling window approach generalises well across different physiological contexts, as prediction performance remains consistent across all PA levels (81.67–84.03%), with only minor variation despite increasing physiological variability at higher PA intensities. In real-world T1DM management, predicting BG changes following PA is particularly challenging, especially at higher intensities where physiological responses are more variable. The ACTIVE-GLU model’s ability to maintain over 80% accuracy across all PA categories shows its potential clinical utility. Accurate prediction of BG changes within a defined variability band enables more informed insulin dose adjustments and carbohydrate intake planning, helping to prevent hypoglycaemia without excessive compensation.

### Scatter plot comparison of predicted and actual glucose changes

All evaluation metrics were computed at the participant level. Each participant had an independently trained rolling-window model, and the distributions therefore represent variability across individuals rather than aggregated cohort-level statistics. To visually assess the correlation between predictions and observed outcomes, we generated scatter plots of predicted versus actual next-day BG changes for each PA intensity level. This was done by calculating the mean predicted and actual BG changes per participant within each PA category to provide a generalised view of model performance. This comparison was carried out in two stages: initially using only the predicted and actual mean values, and subsequently incorporating predicted prediction intervals (expressed as Predicted Mean ± STD) to evaluate model uncertainty.

**Figure 3.**
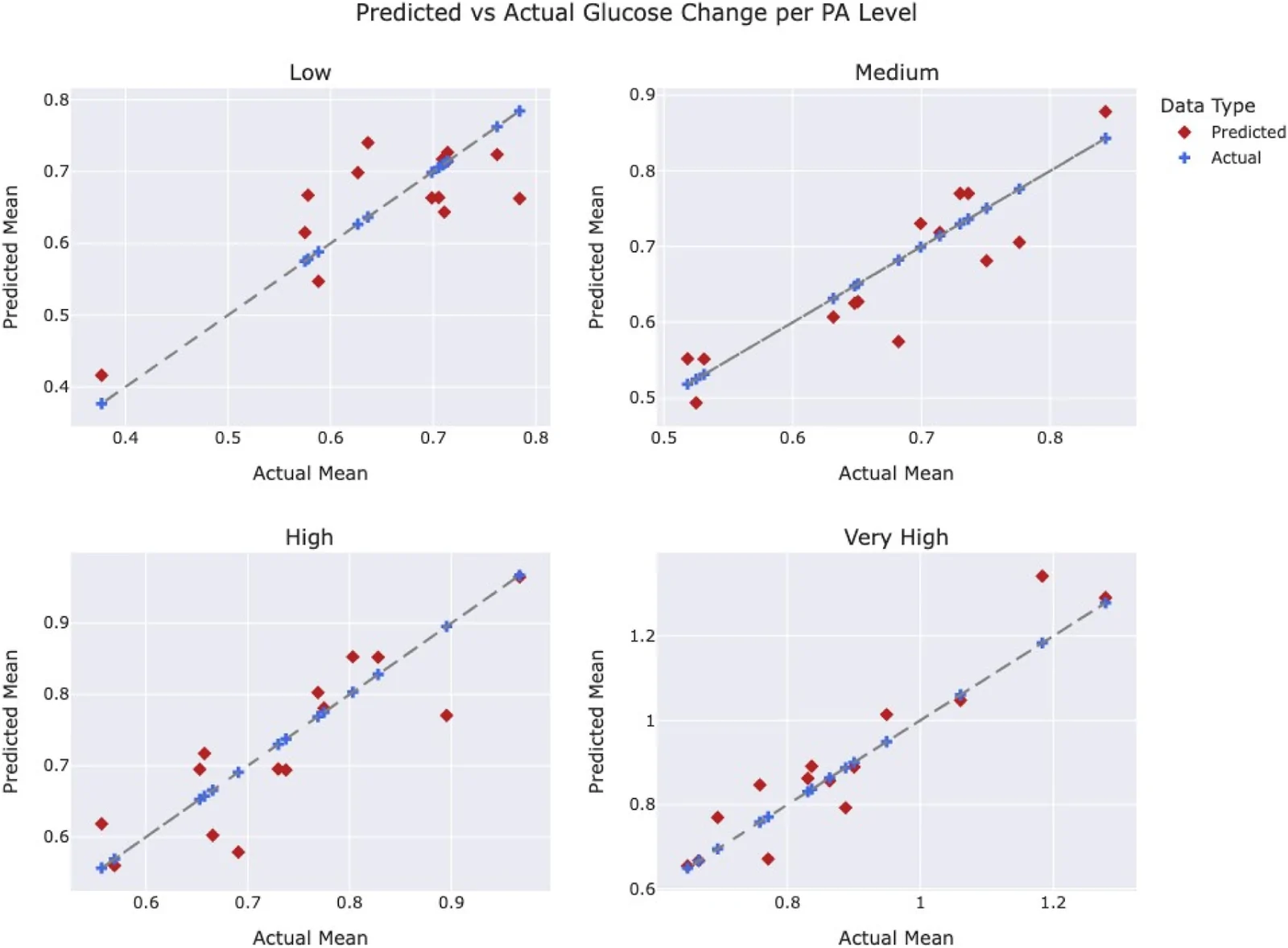
Predicted versus actual next-day glucose changes (Δ*BG*, mmol/L) for each PA intensity level across the study cohort (*n* = 14). Each point represents the mean BG change estimated from an individual participant’s personalised rolling-window model. Blue crosses denote observed mean glucose changes, while red diamonds indicate predicted mean values. The dashed diagonal line (*y* = *x*) represents perfect agreement between predicted and observed glucose changes.

Across all PA levels, the majority of predicted points aligned closely with their corresponding actual values as shown in Figure 3, indicating that the model captured the directional trend of BG change under varying PA intensities. The best agreement was observed in the Low and Medium PA groups, where data points closely followed the y = x line. The High and Very High PA groups showed slightly more scatter, particularly for a few participants whose predictions deviated from the actual observations. However, these deviations are generally clinically insignificant, and the overall pattern shows a consistent predictive association between predicted and actual outcomes.

**Figure 4.**
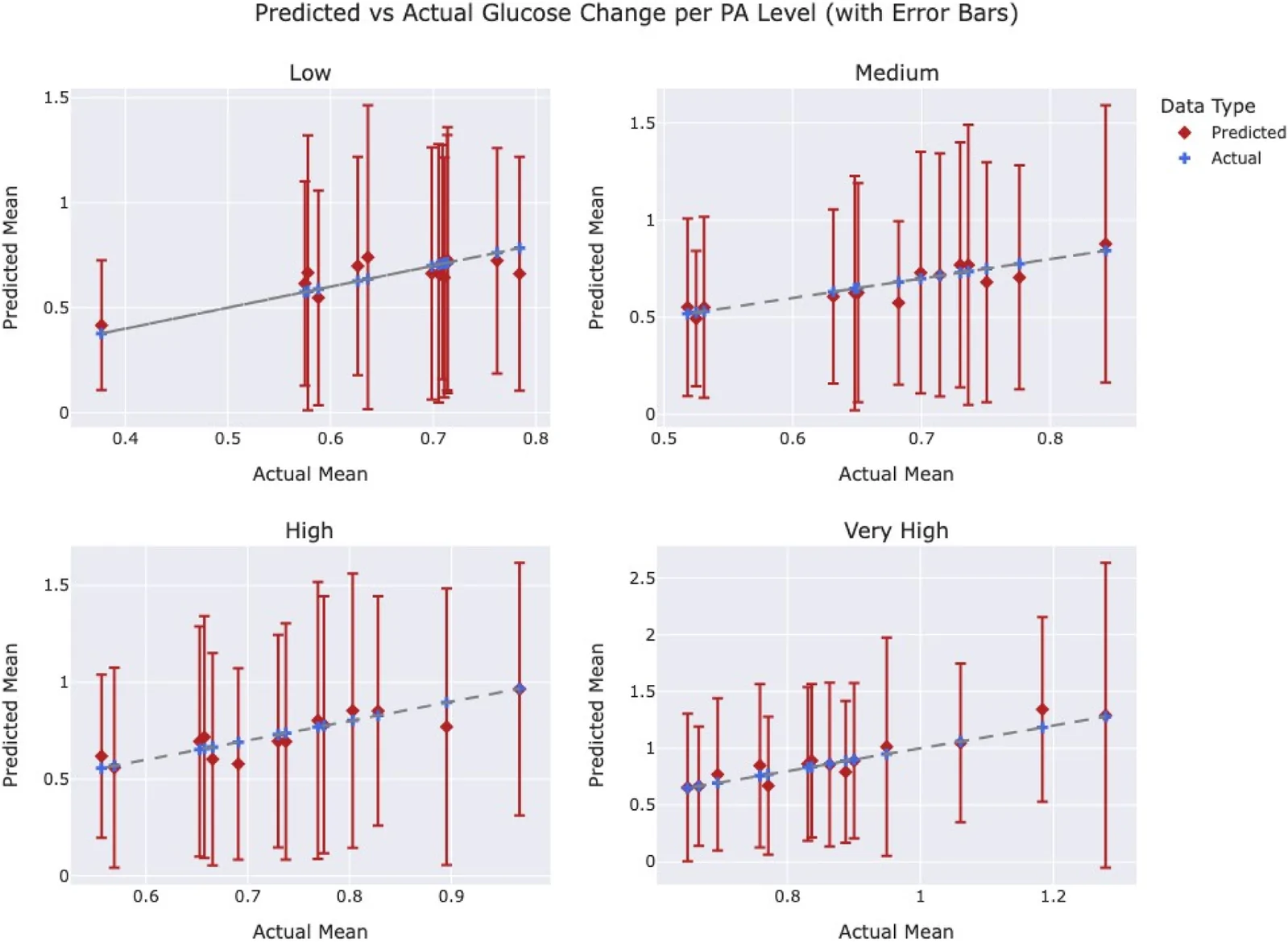
Predicted versus actual glucose changes (Δ*BG*, mmol/L) with prediction intervals across the study cohort (*n* = 14). Each point corresponds to the mean BG change estimated from an individual participant’s personalised rolling-window model. Red diamonds represent predicted mean glucose changes, with vertical error bars indicating the prediction interval (predicted mean ± predicted STD). Blue crosses denote observed mean glucose changes. The dashed diagonal line (*y* = *x*) indicates perfect agreement between predicted and observed values.

To further evaluate the reliability of the model, we extended the analysis by incorporating error bars representing the predicted prediction interval (Predicted Mean ± Predicted STD), as shown in Figure 4. These plots provide insight into how well the model quantifies its uncertainty, especially in the context of physiological variability across PA levels. This reflects the role of the STD term (*Y*) in Equation (5), where the actual BG change was modelled as *X* ± *Y*, with *X* representing the predicted mean and *Y* representing the predicted variability (STD) used to define the upper and lower bounds.

The STD-based error bars were narrowest in the Low and Medium PA categories, reflecting more stable BG change patterns and greater model certainty in those contexts. In contrast, under High and Very High PA levels, the predicted STD increased, suggesting greater physiological variability. The Very High PA level exhibited the widest spread in predicted bounds, aligning with the broader distributions observed in the Mean Bound Deviation violin plot (Figure 8). Despite this, the majority of predictions across all PA levels remained within clinically acceptable margins.

In cases with wider prediction intervals, most actual values fell within or near the predicted range, supporting the model’s adaptive estimation of uncertainty. Outlier cases, such as ID2308 in the Very High PA group, exhibited prediction bounds of 1.89 mmol/L, but this deviation occurred in only one window out of ten. Taken together, these visualisations demonstrate that the model not only provides accurate point predictions but also adaptively quantifies its uncertainty across diverse PA intensities, supporting its reliability and interpretability in real-world BG forecasting.

**Figure 5.**
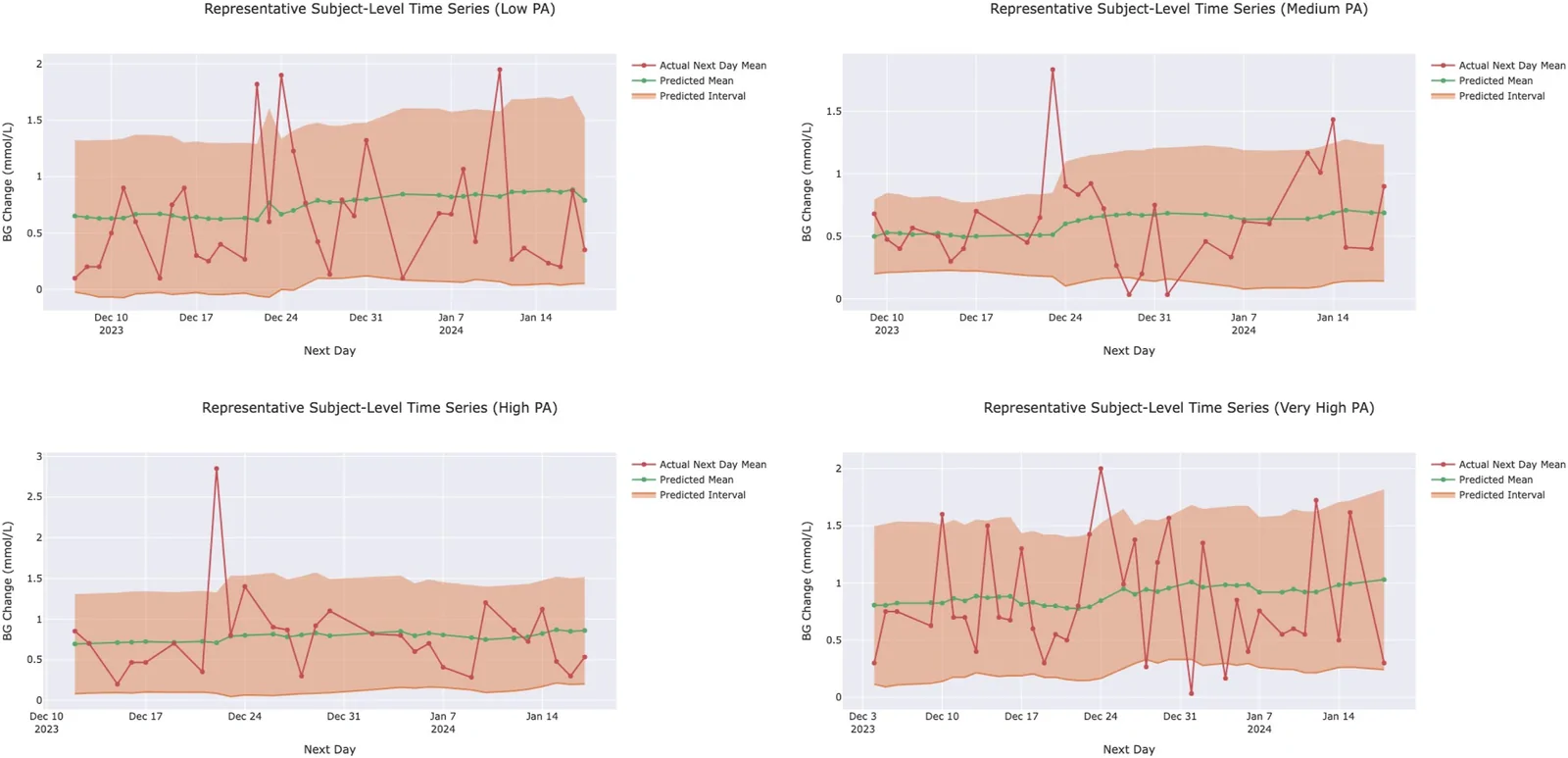
Representative subject-level time-series plots illustrating predicted and observed next-day BG changes across sequential rolling windows for a participant (ID2301) under four PA intensity levels: (a - top left) Low, (b - top right) Medium, (c - bottom left) High, and (d - bottom right) Very High PA. The green line represents the predicted mean BG change derived from the personalised rolling-window model, while the shaded region denotes the prediction interval defined by the predicted mean ± predicted STD. The red line indicates the observed next-day BG change. These plots demonstrate the personalised nature of the modelling framework and illustrate how prediction uncertainty adapts to increasing physiological variability at higher PA intensities.

To further illustrate the personalised nature of the proposed rolling-window framework, we present representative subject-level daily time-series plots showing predicted and observed BG changes across sequential windows for participant ID2301 under different PA intensity levels Figure 5. The predicted mean BG change evolves gradually over time, reflecting the stabilising effect of the historical window, while the observed BG responses exhibit greater short-term variability. Across most windows, the observed BG change falls within the predicted interval (mean ±STD), indicating that the model captures the expected physiological variability associated with PA. As PA intensity increases, the variability of observed responses becomes more pronounced, particularly in the Very High PA level, and the prediction intervals widen accordingly. This behaviour shows the model’s ability to adapt its uncertainty estimates to changing physiological conditions while maintaining a consistent predictive trend.

#### Error metrics

To complement accuracy, we examined three additional error metrics across all PA levels: MAE, RMSE, and Mean Bound Deviation. These metrics quantify not only the magnitude of prediction errors but also the stability and spread of the predicted BG deviations under varying PA levels. We also computed the generalised mean of MAE, RMSE, and Mean Bound Deviation for each PA intensity level. These aggregated figures provide a broader perspective on model performance and its ability to generalise across individuals within each PA category. The summarised metrics are as follows:

• **Low PA:** MAE = 0.36 mmol/L, RMSE = 0.48 mmol/L, Mean Bound Deviation = 0.35 mmol/L
• **Medium PA:** MAE = 0.35 mmol/L, RMSE = 0.47 mmol/L, Mean Bound Deviation = 0.33 mmol/L
• **High PA:** MAE = 0.39 mmol/L, RMSE = 0.52 mmol/L, Mean Bound Deviation = 0.41 mmol/L
• **Very High PA:** MAE = 0.44 mmol/L, RMSE = 0.57 mmol/L, Mean Bound Deviation = 0.38 mmol/L

**Figure 6.**
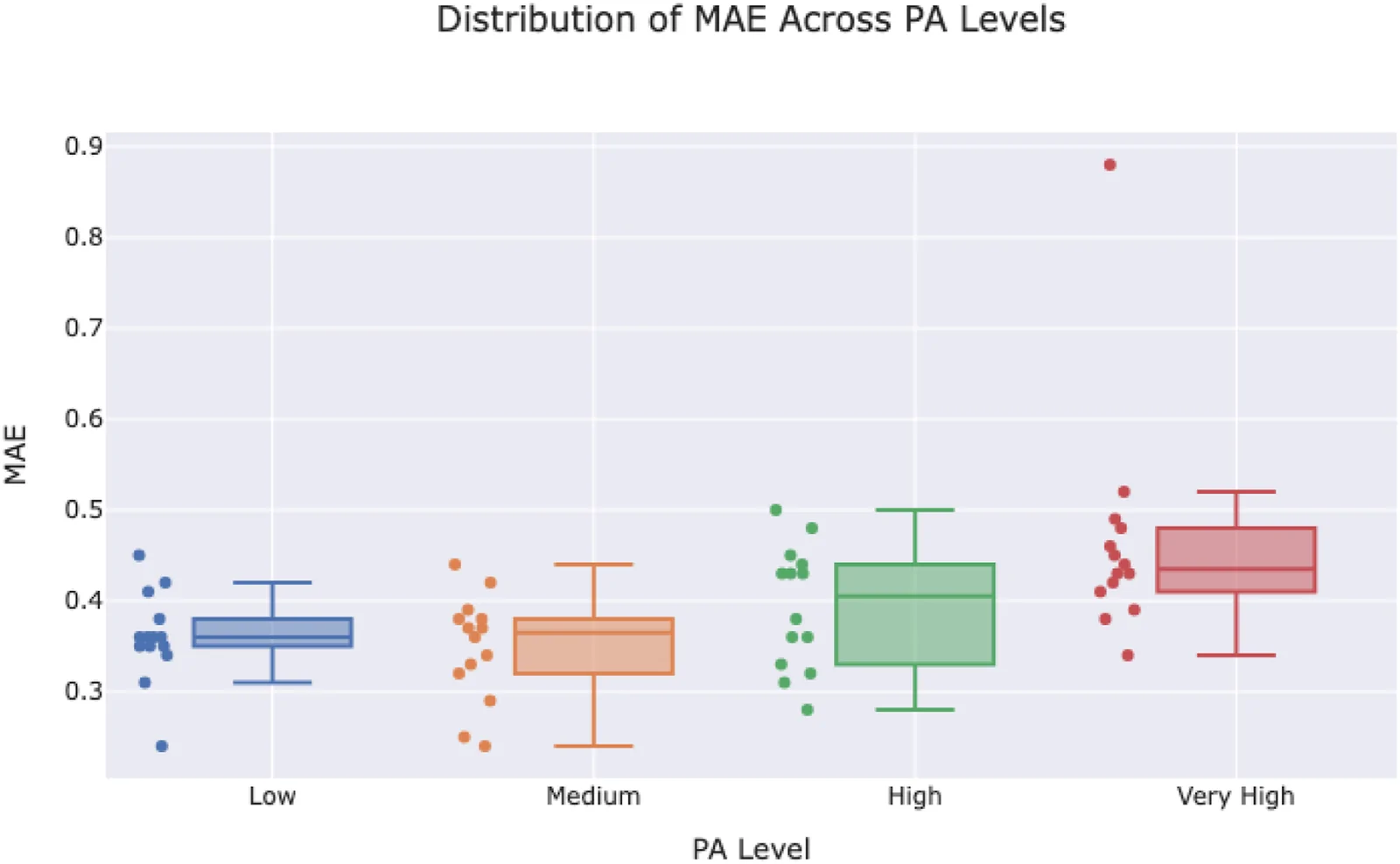
Distribution of participant-level mean absolute error (MAE, mmol/L) across participants (*n* = 14) for each activity category. MAE remained below 0.5 mmol/L for most participants, with narrower distributions observed in the Low, Medium, and High PA categories. Each MAE value represents the prediction error obtained from an individual participant’s personalised rolling-window model.

**Figure 7.**
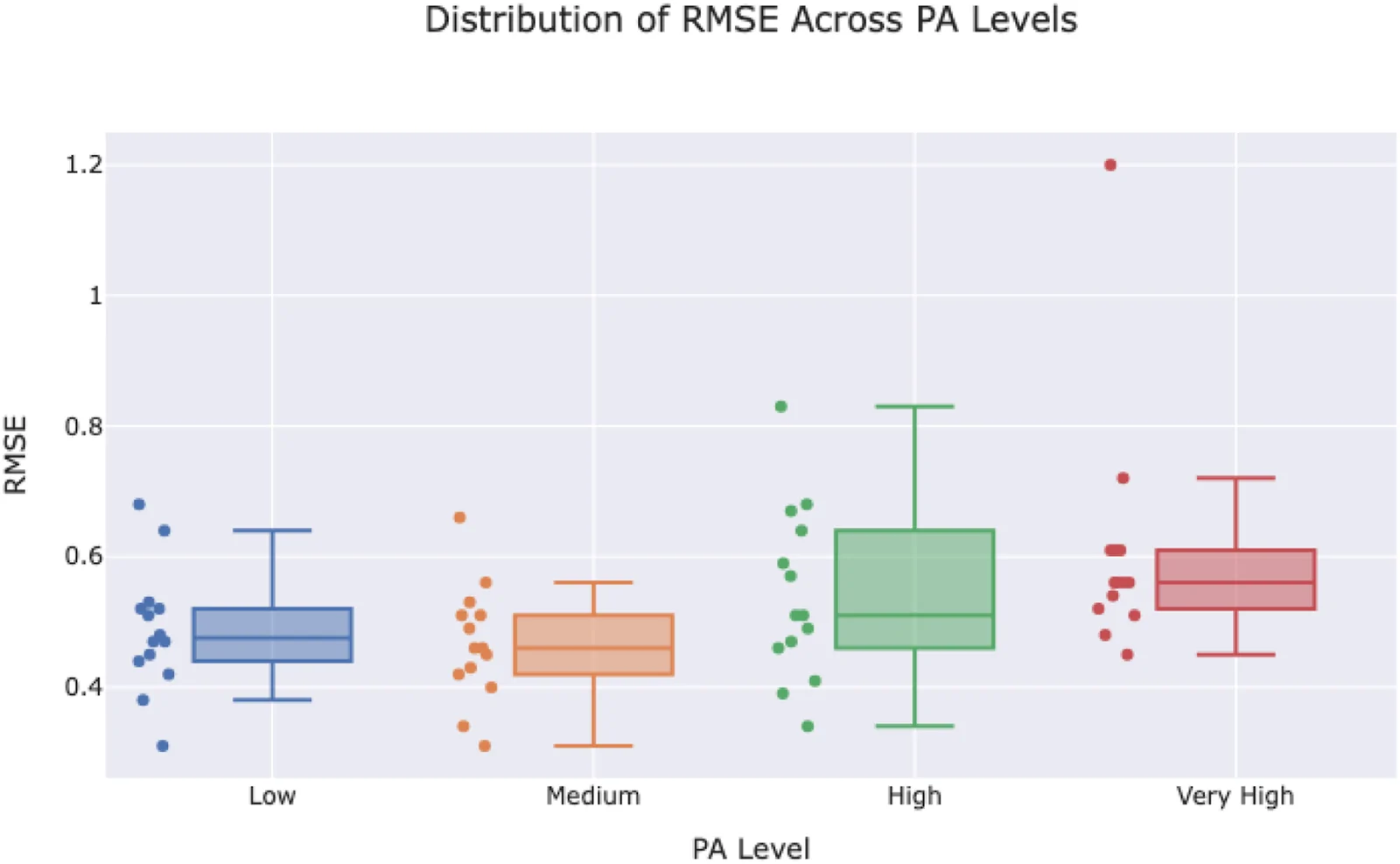
Distribution of participant-level root mean squared error (RMSE, mmol/L) across PA categories for the study cohort (*n* = 14). Higher PA intensities show slightly wider RMSE distributions, reflecting increased variability in glucose responses during intense activity. Each RMSE value corresponds to prediction error derived from an individual participant’s personalised rolling-window model.

These generalised metrics confirm that the model maintained consistent predictive association performance across PA levels. Although errors slightly increased with PA intensity, as expected due to greater BG variability, they remained well within clinically acceptable thresholds. The Mean Bound Deviation values further indicate that, when interval violations occurred, the magnitude of deviation beyond the predicted bounds was generally small. As shown in Figures 6 and 7, both MAE and RMSE remained within clinically acceptable limits across all four PA levels.

Under Low and Medium PA conditions, the model consistently produced lower errors, with median MAE values near 0.35 mmol/L and RMSE values near 0.48 mmol/L. These distributions were tight, indicating that most predictions were closely aligned with actual BG changes. In low PA Table 3 and ID2320, despite having the lowest accuracy, still showed a MAE of just 0.24 mmol/L and RMSE of 0.31 mmol/L, suggesting that misclassifications were minor in magnitude. In contrast, High and Very High PA conditions introduced more variability, as evidenced by slightly elevated medians and wider interquartile ranges in both MAE and RMSE box plots. This is expected due to the physiological complexity and unpredictable BG dynamics under intense PA. Nevertheless, the model continued to perform consistently, with only one outlier RMSE values exceeding 1 mmol/L.

We observed instances where mean bound deviations approached the predicted BG values, particularly during high or very high PA periods. This was evident for participants ID2301 and ID2308 in the High and Very High PA categories, respectively. Such variations may be explained by limited data points in higher PA categories or the absence of consecutive days of high-intensity activity.

**Figure 8.**
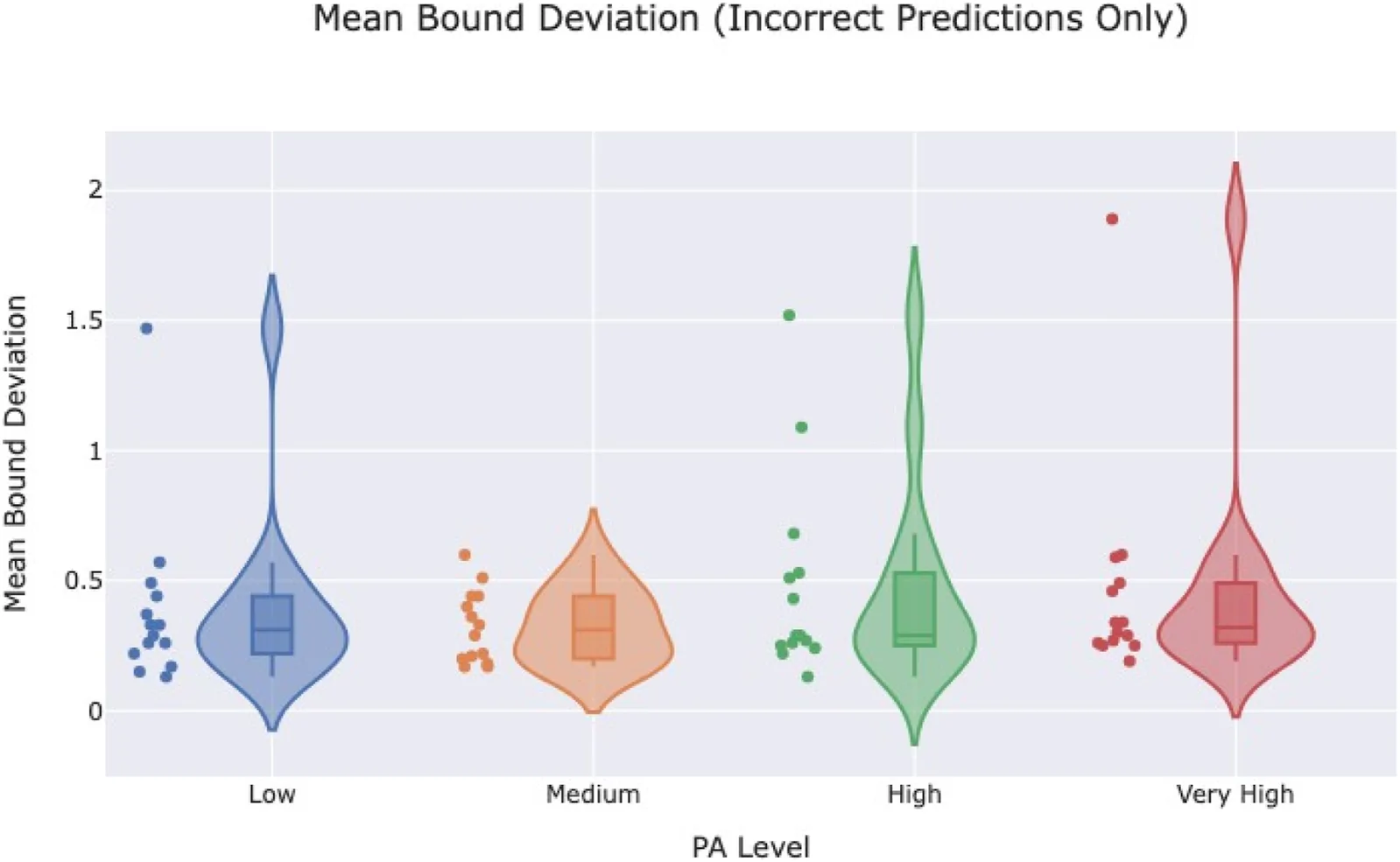
Violin plot showing the distribution of participant-level mean bound deviation (mmol/L) across PA intensities for the study cohort (*n* = 14). Wider distributions of High and Very High PA levels indicate greater prediction uncertainty, consistent with greater physiological variability during higher-intensity PA. Each distribution reflects deviations from predictions derived from individual participants’ personalised rolling-window models.

Mean Bound Deviation quantifies the average magnitude by which observed BG changes fall outside the predicted interval (Predicted BG Change ± Predicted STD), considering only windows in which the observed value lies beyond the predicted bounds. A lower value, therefore, indicates that, when prediction interval violations occur, they are small in magnitude, whereas a higher value indicates larger deviations beyond the predicted range. As illustrated in Figure 8, Mean Bound Deviation remained generally low across PA levels, with median values around 0.3-0.4 mmol/L, suggesting that most interval violations were small. Slightly higher values under High and Very High PA indicate that when prediction errors occurred at higher intensities, the observed BG changes tended to deviate further beyond the predicted bounds. ID2308 in the Very High PA category exhibited a larger Mean Bound Deviation of 1.89 mmol/L, consistent with elevated RMSE; however, this reflected a single out-of-bound event among ten windows.

MAE, RMSE, and Mean Bound Deviation were used together because each captures a different aspect of prediction performance. MAE measures the average absolute difference between predicted and observed BG changes, providing an estimate of overall prediction error. RMSE also measures prediction error but gives greater emphasis to larger deviations between predicted and observed values, making it more sensitive to larger discrepancies. In contrast, Mean Bound Deviation evaluates the average distance by which observed BG changes fall outside the predicted interval defined by the predicted mean and its associated variability, considering only those windows where the observed value lies beyond the predicted bounds. Together, these metrics provide an assessment of model performance by quantifying typical prediction error (MAE), sensitivity to larger deviations (RMSE), and the magnitude of prediction-interval violations (Mean Bound Deviation).

### Statistical comparisons

**Figure 9.**
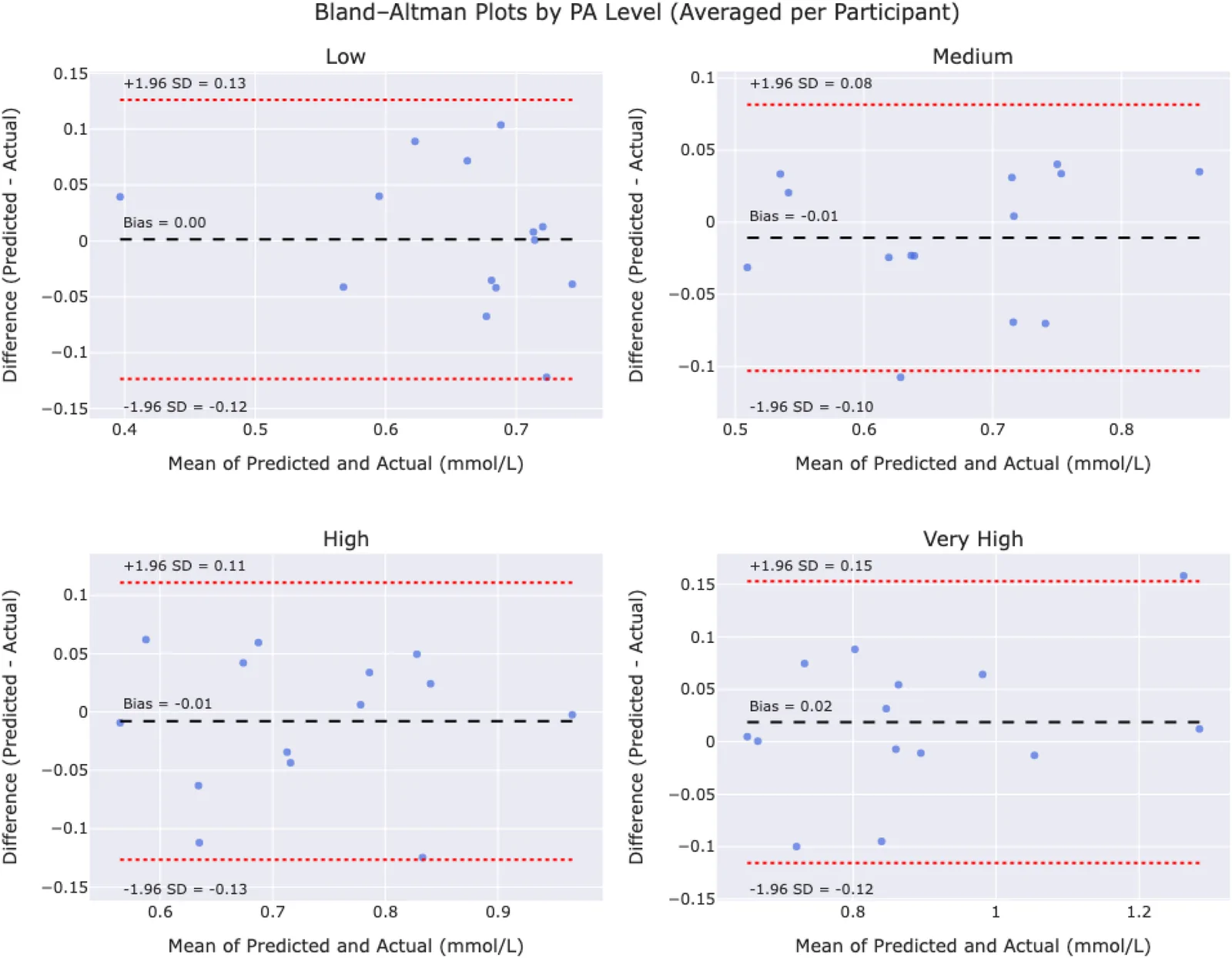
Bland-Altman plots illustrating the agreement between predicted and actual BG changes (Δ*BG*, mmol/L) across participants (*n* = 14) in four PA categories (Low, Medium, High, and Very High). Each plot shows the differences between predicted and actual BG (y-axis) versus their averages (x-axis). The mean difference (green dashed line) represents the average bias. In contrast, the red dotted lines denote the upper and lower limits of agreement (±1.96 standard deviations), within which approximately 95% of the differences are expected to lie. Outliers above or below these limits highlight rare discrepancies possibly due to individual variability in BG.

Bland–Altman analysis^52^ was used to assess agreement between predicted and observed BG changes. This method evaluates the difference between two measurements relative to their mean, allowing identification of systematic bias and estimation of the limits of agreement (LoA), within which 95% of differences are expected to lie. Across all PA levels, mean differences were close to zero, indicating minimal systematic bias, and most observations fell within the 95% LoA as shown in Figure 9.

In the Low PA group, the mean bias was 0.00 mmol/L, with LoA ranging from –0.12 to 0.13 mmol/L, and a symmetric distribution around the bias line. The Medium PA group showed similar agreement, with a bias of –0.01 mmol/L and narrower limits (–0.10 to 0.08 mmol/L), although one observation slightly exceeded the lower bound. At High PA levels, the bias remained low (–0.01 mmol/L), with LoA from –0.13 to 0.11 mmol/L, and no clear outliers observed. In the Very High PA group, variability increased slightly, with a bias of 0.02 mmol/L and a LoA of –0.12 to 0.15 mmol/L; one observation exceeded the upper bound. Overall, the results indicate good agreement between predicted and observed BG changes across PA levels, with only minor deviations in higher-activity conditions.

### External validation on the HUPA-UCM dataset

To further evaluate the generalisability of ACTIVE-GLU beyond the original study cohort, the model was applied without modification to the publicly available HUPA-UCM dataset,^53^ an independent multimodal T1DM dataset collected at Hospital Universitario Príncipe de Asturias, Alcalá de Henares, Spain. This dataset comprises CGM readings obtained via Abbott FreeStyle Libre 2 devices (15-minute intervals), alongside step counts and continuous heart rate data recorded using Fitbit Ionic smartwatches, from 25 individuals with T1DM under free-living conditions (mean age 39.2 ± 11.8 years; 52% female; 56% CSII, 44% MDI).

### Dataset comparison and cohort qualification

It is important to contextualise the HUPA-UCM dataset relative to the primary dataset used in this study.^31^ The primary dataset provides substantially richer longitudinal coverage: 14 participants each contributed up to 12 weeks of synchronised, high-resolution CGM and wearable PA data, yielding approximately 452,000 raw PA records and 339,000 CGM readings. By contrast, the HUPA-UCM dataset, while comprising 25 participants in total, provided considerably shorter recording windows and more fragmented data series. Of the 25 participants, only 10 met the minimum requirement of at least 10 consecutive days of synchronised PA and BG-decrease intervals across all four PA intensity levels necessary to construct a valid rolling-window model. The remaining 15 participants were excluded due to insufficient data continuity, underscoring the importance of longitudinal data density, a key design advantage of the primary dataset.

Furthermore, among the 10 qualifying participants, only three: HUPA0026, HUPA0027, and HUPA0028, contributed data volumes comparable to those in the primary cohort Table 7 (Low PA), Table 8 (Medium PA), Table 9 (High PA), and Table 10 (Very High PA). The remaining seven participants had substantially fewer prediction windows per PA level (range: 1–4), limiting the reliability of their individual predictions; however, all predictions remained within acceptable bounds. Despite these data constraints, ACTIVE-GLU maintained high performance across the three participants. HUPA0027 contributed the largest external validation set, with a total of 334 prediction windows under the Very High PA category alone, and achieved an accuracy of 78.14%. This result is particularly important, as it shows that the personalised rolling-window framework can maintain meaningful predictive accuracy on a fully independent cohort using a different wearable device and collected in a different country. The reliability of performance under these conditions reflects the adaptive, individualised nature of the ACTIVE-GLU framework, which learns exclusively from each participant’s own historical PA-BG dynamics rather than population-level assumptions.

**Table 7.** Participant-wise prediction performance under the Low PA level. The model provides high predictive alignment for most participants, with several achieving perfect accuracy. Larger cohorts maintain stable performance with moderate error and acceptable bound deviations, indicating reliability under low activity conditions.

Participant ID	Accuracy (%)	Predicted BG Change - Average (mmol/L)	Predicted BG Change - std. Dev. (mmol/L)	Actual next Day Mean (mmol/L)	MAE (mmol/L)	RMSE (mmol/L)	Mean Bound Deviation (mmol/L)	Correct/Total Windows
HUPA0001	100.00	0.79	0.53	1.13	0.34	0.34	0.00	1/1
HUPA0003	50.00	0.50	0.38	0.48	0.26	0.32	0.05	2/4
HUPA0007	100.00	0.76	0.58	0.67	0.09	0.10	0.00	2/2
HUPA0011	66.67	0.69	0.55	1.02	0.33	0.41	0.18	2/3
HUPA0014	100.00	0.45	0.36	0.33	0.12	0.12	0.00	1/1
HUPA0018	100.00	0.59	0.42	0.51	0.17	0.20	0.00	4/4
HUPA0025	100.00	0.49	0.52	0.24	0.25	0.27	0.00	4/4
HUPA0026	83.61	0.59	0.61	0.60	0.34	0.49	0.39	102/122
HUPA0027	80.51	0.58	0.55	0.58	0.35	0.47	0.34	314/390
HUPA0028	83.67	0.62	0.60	0.71	0.47	0.66	0.60	41/49

**Table 8.** Prediction summary for the Medium PA category. Accuracy remains consistently high across participants, with zero bound deviation observed in smaller cohorts. Larger datasets exhibit stable performance with slight variability, showing reliability under moderate activity conditions.

Participant ID	Accuracy (%)	Predicted BG Change - Average (mmol/L)	Predicted BG Change - std. Dev. (mmol/L)	Actual next Day Mean (mmol/L)	MAE (mmol/L)	RMSE (mmol/L)	Mean Bound Deviation (mmol/L)	Correct/Total Windows
HUPA0001	100.00	1.07	0.72	1.00	0.35	0.42	0.00	4/4
HUPA0003	100.00	0.76	0.65	0.49	0.27	0.27	0.00	1/1
HUPA0007	100.00	0.94	0.80	0.56	0.38	0.38	0.00	4/4
HUPA0011	100.00	0.74	0.67	0.56	0.31	0.33	0.00	4/4
HUPA0014	100.00	0.63	0.49	0.44	0.20	0.20	0.00	3/3
HUPA0018	100.00	0.64	0.63	0.57	0.19	0.20	0.00	3/3
HUPA0025	100.00	0.52	0.48	0.27	0.25	0.28	0.00	4/4
HUPA0026	85.47	0.62	0.59	0.65	0.32	0.43	0.31	100/117
HUPA0027	79.59	0.59	0.50	0.59	0.32	0.42	0.24	308/387
HUPA0028	81.13	0.61	0.47	0.62	0.32	0.46	0.42	43/53

**Table 9.** Detailed evaluation for the High PA category. While accuracy remains higher, increased RMSE and bound deviation reflect higher physiological variability during elevated activity levels. Despite this, predictions largely remain within acceptable bounds.

Participant ID	Accuracy (%)	Predicted BG Change - Average (mmol/L)	Predicted BG Change - std. Dev. (mmol/L)	Actual next Day Mean (mmol/L)	MAE (mmol/L)	RMSE (mmol/L)	Mean Bound Deviation (mmol/L)	Correct/Total Windows
HUPA0001	100.00	1.10	0.82	0.91	0.58	0.61	0.00	4/4
HUPA0003	100.00	0.54	0.51	0.55	0.34	0.34	0.00	2/2
HUPA0007	75.00	0.90	0.69	1.04	0.39	0.44	0.13	3/4
HUPA0011	100.00	0.81	0.53	0.78	0.32	0.36	0.00	4/4
HUPA0014	100.00	0.72	0.59	0.60	0.24	0.27	0.00	2/2
HUPA0018	100.00	0.56	0.45	0.44	0.12	0.14	0.00	4/4
HUPA0025	100.00	0.48	0.40	0.31	0.19	0.25	0.00	2/2
HUPA0026	80.51	0.63	0.58	0.66	0.36	0.52	0.43	95/118
HUPA0027	83.07	0.63	0.55	0.64	0.35	0.48	0.39	314/378
HUPA0028	86.21	0.73	0.69	0.72	0.41	0.56	0.45	50/58

**Table 10.** Prediction summary for the Very High PA category. Despite increased activity intensity, the model sustains predictive performance. Some participants exhibit elevated variability and bound deviation, but overall predictions remain within acceptable limits, showing reliability under high PA states.

Participant ID	Accuracy (%)	Predicted BG Change - Average (mmol/L)	Predicted BG Change - Std. Dev. (mmol/L)	Actual Next Day Mean (mmol/L)	MAE (mmol/L)	RMSE (mmol/L)	Mean Bound Deviation (mmol/L)	Correct/Total Windows
HUPA0001	100.00	1.15	0.75	0.77	0.37	0.42	0.00	4/4
HUPA0003	100.00	0.61	0.42	0.71	0.09	0.09	0.00	2/2
HUPA0007	75.00	1.17	0.80	0.79	0.48	0.63	0.35	3/4
HUPA0011	100.00	1.02	0.65	0.74	0.28	0.30	0.00	3/3
HUPA0014	100.00	0.68	0.51	0.59	0.22	0.24	0.00	2/2
HUPA0018	100.00	0.68	0.56	0.51	0.17	0.18	0.00	3/3
HUPA0025	100.00	0.51	0.34	0.46	0.14	0.15	0.00	2/2
HUPA0026	84.21	0.70	0.67	0.73	0.40	0.53	0.50	96/114
HUPA0027	78.14	0.74	0.59	0.76	0.41	0.56	0.35	261/334
HUPA0028	95.45	0.68	0.59	0.67	0.30	0.48	1.06	42/44

#### Composite PA score adaptation

The HUPA-UCM dataset does not include a direct equivalent of the Garmin maxMotion metric. Accordingly, the composite PA score was computed as the product of step count and heart rate, given that heart rate provides a continuous, epoch-level indicator of exertion intensity during PA^54^. This preserves the dual-dimensional structure of the original composite score, encoding both the volume of movement and the physiological intensity of exertion, and was applied consistently across all qualifying participants.

#### Personalised results

Table 7 (Low PA), Table 8 (Medium PA), Table 9 (High PA), and Table 10 (Very High PA) present participant-level prediction metrics for all 10 qualifying participants across the four PA intensity levels. For participants with sufficient data (HUPA0026, HUPA0027, HUPA0028), accuracy ranged from 78.14% to 95.45%, with all MAE values below 0.50 mmolL. Participants with fewer prediction windows frequently achieved 100% accuracy, though these estimates should be interpreted with caution given the limited window counts. Mean Bound Deviation remained low across most participants, indicating that when prediction interval violations occurred, their magnitude was clinically small.

#### Generalised results and comparison with primary cohort

Table 11 presents generalised performance metrics aggregated across all 10 qualifying HUPA participants, and provides a direct comparison with the primary cohort results. ACTIVE-GLU maintained accuracy exceeding 81% across all four PA intensity levels on this entirely independent dataset (81.55%-83.33%), with all MAE values below 0.34 mmol/L. These generalised figures are closely aligned with the primary cohort (81.67%-84.03% accuracy; MAE 0.35–0.44 mmol/L), and MAE on the HUPA dataset was in fact equal to or lower than the primary cohort across every PA level, despite the differences in dataset characteristics, device platform, and PA metric derivation.

**Table 11.** Comparison of generalised prediction performance between the external HUPA-UCM validation cohort (*n* = 10) and the primary study cohort (*n* = 14) across all four PA intensity levels. MAE and RMSE are expressed in mmol/L. Accuracy values are closely aligned across both cohorts while MAE on the HUPA-UCM dataset is consistently equal to or lower than the primary cohort, despite differences in wearable device, PA metric derivation, CGM platform, and participant demographics.

PA level	HUPA-UCM (External)			Primary cohort		
	Accuracy (%)	MAE	RMSE	Accuracy (%)	MAE	RMSE
Low	81.55	0.27	0.34	81.67	0.36	0.48
Medium	81.72	0.29	0.34	84.03	0.35	0.47
High	83.33	0.33	0.40	81.93	0.39	0.52
Very High	81.64	0.29	0.36	82.12	0.44	0.57

Taken together, these findings show that the personalised rolling-window approach of ACTIVE-GLU generalises reliably to unseen individuals, wearable platforms, and recording conditions. The performance across two datasets collected in different countries using different devices, and the particular strength of results for the three HUPA participants with substantial data volumes, supports the view that accurate PA-aware BG prediction in T1DM is achievable under real-world heterogeneity, provided that sufficient longitudinal within-participant data are available. This further shows the design rationale of the primary study dataset,^31^ which was specifically constructed to provide the dense, synchronised, multi-week PA-CGM recordings that enable reliable personalised modelling.

## Discussion

Our study developed and retrospectively evaluated a personalised predictive model designed to estimate BG changes in response to varying levels of PA in individuals with T1DM. This model will be further evaluated through simulation-based testing before potential real-world implementation. Using data from 14 participants in free-living conditions, the model achieved accuracy (>81.67%) at 15-minute intervals, with maximum Mean Bound Deviation (<1.89). Consecutive days of data are required since our rolling window requires at least 10 days of observations. Once this window is established, the model computes the mean BG change and the corresponding variability (STD). After estimating the predicted change in BG levels for the next 15-minute interval using *X* and *Y*, the change can be expressed as *X* ± *Y*, as shown in Equation (5). To account for potential minimum and maximum drops in the next interval, we consider *X* − *Y* and *X* + *Y*, respectively. Here, *Y* represents the STD, indicating variability around the average BG change.^54^ Accordingly, the primary contribution of this study lies in demonstrating the feasibility of modelling PA–BG interactions using wearable-derived data within a personalised rolling-window framework, consistent with time-series modelling approaches that rely on moving averages and adaptive updating.^45,55^

Our approach predicts BG changes at 15-minute intervals following PA, enabling timely estimation of PA-induced BG fluctuations. Because BG responses to PA can occur rapidly due to increased glucose uptake and insulin sensitivity,^3,56^ predictions at 15-minute intervals provide a more responsive framework than approaches relying on longer aggregation windows. Maintaining glucose within clinically recommended ranges requires timely intervention.^4^ Using historical observations, the model estimates the expected magnitude of BG change shortly after a given level of PA. A strength of this study is the richness of the longitudinal dataset. Across 14 participants, we collected approximately 452,000 raw PA records and 339,000 CGM readings. Despite aggregation, the high temporal resolution enabled detailed characterisation of free-living PA–BG dynamics and supported reliable within-participant modelling, which is essential given the high inter- and intra-individual variability in T1DM BG responses.^25,26^

The selection of a rolling-window approach over ML reflects deliberate design constraints. ACTIVE-GLU is intended as a real-time, on-device tool operating without network connectivity, a context in which ML inference architectures are impractical.^17,57^ ML models for BG prediction also require substantially larger training datasets than individual free-living participants typically generate,^58,59^ and existing PA-aware ML approaches have predominantly been evaluated under structured exercise protocols with multimodal inputs,^18,60,61^ limiting direct comparability with the free-living, PA-only framework presented here. The rolling-window algorithm achieves clinically acceptable accuracy (MAE < 1 mmol/L) using only local storage and simple arithmetic, without the data, compute, or connectivity requirements of ML approaches, and is easy for clinicians to interpret. Within-participant longitudinal depth, rather than population breadth, provides the basis for personalisation,^58^ further reducing data requirements relative to population-level models.

Among publicly available CGM datasets,^62^ relatively few include detailed PA information,^53^ and it is particularly uncommon for the precise start time of activity to be explicitly annotated. Recent open CGM datasets have primarily focused on glucose, insulin, and carbohydrate information, with limited contextual data describing activity timing.^63^ In contrast, the dataset^31^ used in this study includes explicit annotations of PA episodes under free-living conditions, enabling more direct analysis of PA-induced glycaemic responses. This is particularly important given that unstructured daily PA has been shown to significantly influence glucose variability and hypoglycaemia risk.^10^

The personalised design of ACTIVE-GLU has implications for generalisability. Rather than relying on cohort-level assumptions, the model adapts to each individual by accounting for longitudinal PA–BG interactions, consistent with prior work highlighting substantial inter- and intra-individual variability in T1DM.^57,58^ While the cohort consisted of 14 participants, each contributed dense longitudinal observations, enabling within-participant modelling based on repeated PA–BG interactions rather than single cross-sectional samples. This approach aligns with evidence that reliable BG characterisation can be achieved using multi-day CGM data.^49^ Consequently, effective personalisation depends on sufficient longitudinal coverage rather than large population cohorts. However, the limited cohort size restricts the evaluation of inter-individual variability and broader external validity. Additionally, specific physiological states such as pregnancy, paediatric diabetes, or elite athletic training may not be fully represented.^64–66^ Future studies with larger and more diverse populations are therefore required.

ACTIVE-GLU was developed as an adaptive, personalised framework that learns from each participant’s historical physiology and behaviour rather than applying population-level thresholds. Consequently, PA intensity was characterised relative to each participant’s personal baseline rather than fixed absolute values, consistent with evidence that physiological responses to PA depend on individual fitness, insulin sensitivity, and habitual activity levels.^14,67^ The mean daily PA level served as an individualised reference defining non-standard activity relative to habitual behaviour. For sedentary individuals, modest increases in movement may represent meaningful exertion, whereas for highly active individuals, similar absolute intensities may fall within baseline variability. By continuously updating using preceding data windows, the model integrates recent behaviour and physiological state, enabling day-to-day adaptation under free-living conditions. This is particularly important given the known dissociation between relative intensity (individual physiological strain) and absolute intensity (external workload), where measures such as heart rate may not accurately reflect underlying metabolic demand over time.^68^

Previous studies have shown that incorporating exercise-related information can enhance BG prediction accuracy.^69,70^ BG prediction has been investigated in controlled exercise settings,^18,61,71^ clinical trials,^60,72^ and real-world monitoring environments,^73–75^ as well as through in silico simulations.^76,77^ However, many approaches rely on structured exercise protocols or multimodal inputs including insulin and meal data, which may be inconsistently recorded in free-living conditions and introduce additional uncertainty.^27^ In contrast, ACTIVE-GLU focuses specifically on wearable-derived PA and CGM data to isolate the direct effect of activity on BG dynamics, addressing a gap in modelling spontaneous daily activity. It is acknowledged that this design choice may introduce confounding, as unrecorded compensatory carbohydrate intake or insulin adjustments around PA episodes could cause fluctuations in observed BG responses, limiting associated interpretation; future work will incorporate these variables once reliable automated synchronisation becomes feasible.^26,27^

Our study utilises accuracy percentages and MAE to deliver BG predictions at 15-minute intervals with minimal error, demonstrating the potential to support real-time diabetes management. Moreover, it focuses specifically on BG and PA data and provides predictions at shorter 15-minute intervals, allowing more rapid responses to activity-induced changes while avoiding the complexity of integrating multiple behavioural variables. The study^18^ used models, such as Multivariate Adaptive Regression, to predict BG levels during and after exercise, achieving sensitivities of approximately 63–71% and accuracies of up to 81%. In comparison, our study predicts BG changes in real-world free-living conditions with accuracy ranging from 81.67% to 84.03% at 15-minute post-activity intervals. This approach broadens applicability beyond structured exercise to everyday PA, supporting more continuous monitoring of BG dynamics.

The model also quantifies both the expected BG change and its variability using STD. In statistics, STD measures the spread of values around the mean and therefore provides insight into data consistency.^54^ In ACTIVE-GLU, the STD defines the predicted bounds around the expected BG change for each level of PA. The rolling window was maintained for *n* days because physiological responses to PA evolve over time and reliable characterisation of BG patterns generally requires repeated observations collected across multiple days.^49^ Evidence describing PA-related changes in insulin absorption and BG dynamics supports the importance of monitoring physiological adaptation over multiple days.^78^ In our study, the average BG drop associated with each PA level stabilised after approximately 10–13 days, depending on the participant. For some individuals, the STD stabilised after 10 days, whereas for others it stabilised after 13 days, which determines the effective rolling-window size.

The clinical applicability of ACTIVE-GLU varies across insulin delivery modalities. For individuals using open-loop or manual regimens such as MDI or non-automated pumps, the model can provide proactive decision-support information by guiding pre-activity carbohydrate intake or bolus reduction. For users of hybrid or fully closed-loop systems that primarily adjust basal insulin based on current BG trends, ACTIVE-GLU may complement these algorithms by providing anticipatory, PA-aware forecasts. Current closed-loop systems primarily adjust insulin delivery in response to CGM-derived glucose trends and generally lack the ability to automatically detect the onset or intensity of PA. Consequently, exercise management often requires the user to plan ahead, and incorporating PA-aware signals may enhance anticipatory capability.^79^

The framework is designed to operate on a mobile device without network connectivity, reflecting the practical reality that individuals may engage in PA in outdoor settings where internet access is unavailable.^17^ The rolling-window algorithm requires only local storage of recent PA-BG observations and simple arithmetic, making real-time on-device execution feasible without the substantial computational resources and large training datasets required by ML approaches.^57,58^ Rather than generating a single long-horizon forecast, the model is intended to re-predict at regular intervals during ongoing activity, ensuring recommendations reflect the current physiological state. The one-hour prediction window is grounded in the pharmacokinetics of rapid-acting insulin analogues, which reach peak absorption approximately 60 minutes after subcutaneous administration^78^; predicting beyond this horizon without recalibration introduces avoidable clinical risk, particularly in safety-critical dosing decisions.^14^

ACTIVE-GLU could potentially be integrated into decision-support tools such as adaptive bolus calculators^30^ or mobile health applications.^80^ In such systems, predictions could provide interpretable information about expected short-term BG movement and physiological uncertainty. These estimates could support preventive carbohydrate intake or modest bolus adjustments when PA is anticipated, although further validation would be required before clinical deployment. To further evaluate the model’s reliability, we plan to validate ACTIVE-GLU using a virtual patient simulator.^81^ This platform enables in silico testing of glycaemic responses in individuals with T1DM under varying combinations of PA, insulin dosing, and meal intake. The simulator supports multiple therapy modes, including MDI and hybrid closed-loop systems, and provides access to a diverse virtual patient population. This approach will allow external evaluation of the prediction framework using simulated participants while enabling the exploration of higher PA scenarios and uncertainty in PA timing.^82^

Although evaluated retrospectively, ACTIVE-GLU operates as a rolling-window system that continuously adapts to behavioural and physiological changes. When patterns shift, prediction variability may temporarily increase; however, adaptive updating enables realignment with the evolving physiological state. Because predicted BG changes remain relatively small (typically within ± 1 mmol/L), any future translation into carbohydrate or insulin recommendations would involve modest adjustments rather than large corrective actions. As such, the model provides estimates of the expected direction and magnitude of BG change rather than directly prescribing insulin or carbohydrate adjustments. These predictions may support safer decision-making in free-living environments, particularly for individuals relying on anticipating BG trends during daily activities.

Our study focuses on predicting BG changes at 15-minute intervals following PA, potentially overlooking cumulative responses during prolonged activity. This is reflected in Figure 6, where BG STD MAEs were higher under High, and Very High PA levels, indicating increased variability is consistent with delayed and nonlinear glycaemic responses during intense or prolonged exercise.^9,13^ Nevertheless, the magnitude of error remained small, with STD MAEs not exceeding 0.35 mmol/L, which remains within clinically acceptable bounds. Future research could extend the prediction horizon or apply multi-step forecasting approaches to capture delayed glycaemic dynamics.

### Limitations

ACTIVE-GLU was designed specifically to model downward BG excursions following PA. This choice reflects clinical evidence that low-to-moderate intensity PA often produces BG declines shortly after activity onset, representing the most immediate hypoglycaemia risk. High-intensity PA responses are more variable because counterregulatory responses may cause BG to decrease, remain stable, or increase. This variability likely contributed to the higher prediction errors observed in the High and Very High PA categories. Future work should therefore expand the framework to capture neutral and upward BG responses in order to improve accuracy across a wider range of activity intensities.

## Conclusion and Future work

This exploratory study demonstrates a consistent predictive association between PA and BG levels in individuals with T1DM. By quantifying PA and using personalised predictive models, we achieved high accuracy in forecasting BG fluctuations under free-living conditions. These findings establish a methodological foundation for PA-aware predictive modelling and support future work on simulation-based translation of predicted BG changes into insulin and carbohydrate adjustment strategies. Future research will translate these predictions into insulin and carbohydrate adjustments by mapping estimated BG changes to each individual’s carbohydrate-to-insulin ratio (CIR) and correction factor (CF).^30^

(8) $${\text{Insulin}}_{\text{adj}}=\frac{\Delta BG_{\text{pred}}}{CF},$$

(9) $${\text{CHO}}_{\text{req}}={\text{Insulin}}_{\text{adj}}\times CIR.$$

Implementing this stage within a simulation framework will allow safe *in silico* testing before real-world deployment. Therefore, ACTIVE-GLU may function as an adaptive PA-aware decision-support system for open-loop insulin delivery and as a supportive module within hybrid closed-loop systems.

## Data Availability

The dataset and source code for this study are available at Primary Repository and HUPA Repository. This study used Python 3.8.8, developed with Anaconda and Jupyter Notebook as the programming environment. Researchers are encouraged to access the repository for detailed information.*

## Appendix

### Input

**Table 12.** Raw PA data showing step counts and maxMotion intensity recorded at the same timestamp across multiple activity types.

Timestamp	ActivityType	Step Count	maxMotion
16:30:00	Walking	74	4
16:30:00	Generic	0	7
16:30:00	Sedentary	0	6
16:45:00	Walking	12	2
16:45:00	Sedentary	0	3

### Output

**Table 13.** Aggregated PA score calculated by multiplying step count and maxMotion for each activity type, then averaging across activities per timestamp.

Timestamp	Activity
16:30:00	98.67
16:45:00	12

### Data aggregation and classification

Two key metrics were prioritised in processing the PA data: *step count* and *maxMotion*. To derive a composite PA score at each timestamp, the step count was multiplied by the maxMotion. This approach produces a continuous measure that reflects both the amount and exertion level of movement, enabling sensitivity to different PA intensities. This process is detailed in the Supplementary Information in Algorithm 1. The variable *maxMotion* represents the maximum motion intensity detected within a given epoch, derived from accelerometer data. Motion intensity reflects the magnitude of body movement and provides an additional indicator of PA intensity beyond step counts^82^.

Meanwhile, raw PA data were classified into four states by Garmin’s automatic detection algorithms: Walking, Running, Generic, and Sedentary. Generic denotes movement that does not match predefined labels like indoor cycling, vigorous chores, while Sedentary denotes minimal movement^40^. For each 15-minute epoch, step counts and maxMotion values were aggregated across all detected states to produce a single representative PA value per timestamp (illustrated in Table 12 and Table 13). As only one classification can be retained per epoch, we derived a unified epoch-level PA measure from these aggregated values. Each epoch was then assigned to one of four PA intensity levels (low, medium, high, very high) using participant-specific quantiles.

### Input

**Table 14.** Subset example of raw BG and PA data with timestamps from a CSV file.

Timestamp	Glucose	Activity
17:30:00	7.1	48.2
17:35:00	7.9	-
17:40:00	7.5	-
17:45:00	6.7	70.5

### Output

**Table 15.** Filtered data retaining only intervals with valid BG and PA measurements.

Timestamp	Glucose	Activity
17:30:00	7.1	48.2
17:45:00	6.7	70.5

Basal insulin is typically prescribed to meet the body’s background insulin requirements during routine daily PA^14^. However, PA levels that exceed an individual’s usual exertion require special consideration, as they may increase the risk of hypoglycaemia unless accompanied by appropriate adjustments to insulin or carbohydrate intake^68^. In this study, PA data were classified into two categories: *standard PA*, referring to activity levels below the participant’s mean daily PA, and *non-standard PA*, denoting activity levels exceeding this average. Standard PA generally reflects habitual, lower intensity movements that are well compensated by basal insulin. In contrast, non-standard PA captures deviations from the individual’s regular episodes of higher intensity or longer duration that may precipitate glycaemic fluctuations.

Therefore, we calculated the mean PA level by averaging all PA values recorded across the available 15-minute intervals. Data points with PA values greater than or equal to this mean were selected for further analysis. Research from study^7^ indicates that varying levels of PA can lead to distinct changes in BG levels, influenced by both the timing and intensity of the PA. To facilitate our analysis, we created quantiles of non-standard PA and categorised them into four levels: Low, Medium, High, and Very High PA. These quantiles were individualised, computed separately for each participant from their own non-standard PA distribution to account for differences in habitual activity patterns. Specifically, the quantiles were determined using the following ranges: values from the minimum PA data point to the 25th percentile were classified as Low PA, the 25th to 50th percentiles as Medium PA, the 50th to 75th percentiles as High PA, and the 75th percentile to the maximum PA points as Very High PA, as illustrated in Algorithm 2 in the Supplementary Information.

This quantile-based classification was chosen to ensure an even distribution of PA data across four groups, enabling meaningful comparisons of PA intensity levels within the dataset. As there are no universally accepted cut-off points for PA intensity based on step count and motion data in free-living conditions, quantiles provide a consistent, data-driven method to categorise PA levels. This approach enables a structured investigation of how relative increases in PA intensity are associated with changes in BG dynamics.

The processing of wearable-derived PA data from the Garmin Forerunner 45, including aggregating step count and maxMotion into a composite activity score and subsequently quantile-based classification, constitutes the initial stage of the ACTIVE-GLU. This processed PA signal serves as a key input to the overall predictive pipeline. Classification of PA based on different levels after aggregating non-standard PA data is further formalised in Equation (10).

(10)
$$\begin{array}{c} \text{PA Levels}=\left\{\begin{array}{cc} \text{low}, & \text{if Activity.min}\left(\right)\leq PA\\ & \;<\text{Activity.quantile}\left(0.25\right)\\ \text{medium}, & \text{if Activity.quantile}\left(0.25\right)\leq PA\\ & \;<\text{Activity.quantile}\left(0.5\right),\\ \text{high}, & \text{if Activity.quantile}\left(0.5\right)\leq PA\\ & \;<\text{Activity.quantile}\left(0.75\right),\\ \text{very high}, & \text{if Activity.quantile}\left(0.75\right)\leq PA\\ & \;\leq \text{Activity.max}\left(\right) \end{array}\right. \end{array}$$

Where:

(11)
$$\begin{array}{ll} \text{Activity.min}\left(\right) & \;\text{is the minimum PA level.}\\ \text{Activity.quantile}\left(q\right) & \;\text{is the }q-\text{th quantile of the PA.}\\ \text{Activity.max}\left(\right) & \;\text{is the maximum PA level.} \end{array}$$

We explore the relationship between different levels of PA quantified through step count and maxMotion (step count measures the volume of movement, while maxMotion estimates the peak intensity of movement within each 15-minute epoch) and their corresponding impact on BG changes after 15-minute intervals. We undertook this approach because research^7,14^ has demonstrated that the influence of PA on BG initiates approximately 15-20 minutes into the PA, contingent on the intensity, and can extend for up to 18-24 hours, depending on the intensity.

After identifying the different levels of PA, the next step was to prepare the BG data. Following the process in Algorithm 3 in the Supplementary Information, the CGM-derived glucose data were cleaned by removing missing or duplicated entries and retaining only valid, time-stamped sensor glucose (SG) readings. No temporal smoothing, low-pass, or non-causal filtering was applied at any stage. Each device provided readings at 5–15-minute intervals. To create a uniform series, timestamps were rounded to the nearest 5 minutes and then resampled to 15-minute epochs using the first available SG value at or before each 15-minute boundary. This represents causal undersampling rather than averaging or interpolation, ensuring that every retained value depends only on past information available at that time point.

For each 15-minute interval, the change in glucose concentration was computed as

(12)
ΔBG(t)=SG(t)−SG(t+15min),

So that Δ*BG*(*t*) > 0 indicates a decrease in BG over the next 15 minutes. Rows were retained only where consecutive readings were exactly 15 minutes apart to ensure temporal consistency and accurate alignment with PA data.

### Data Merging

While merging PA and BG data for analysis, inconsistencies were observed across fixed time intervals, with missing BG measurements in some periods and PA measurements in others. Only complete, time-aligned intervals were retained to maintain the accuracy and reliability of the analysis. In this context, data aggregation refers to aligning and merging datasets into uniform time epochs, rather than performing temporal filtering or smoothing. This step ensures temporal consistency between signals, reduces duplication, and improves interpretability for subsequent predictive modelling^43,44^. Table 14 in the Supplementary Information presents an example subset of the combined BG and PA data, highlighting instances where data were missing. Meanwhile, Table 15 in the Supplementary Information shows the filtered dataset after removing incomplete entries. Our study investigates the impact of PA on BG dynamics, focusing exclusively on data from active and resting states while excluding sleep periods. To ensure consistency across participants, we filtered the data to include only entries recorded between 6:00 a.m. and midnight, covering the entire waking day.

The next step is to combine the cleaned and resampled datasets. Algorithm 4 in the Supplementary Information outlines the process of merging the PA data and BG data based on the same “Timestamp” column. After the merge, rows with any missing values are excluded. In addition, the merged dataset is filtered to retain only the rows where the current BG value exceeds the next BG value. This approach predicts BG drops associated explicitly with PA, ensuring the model’s accuracy. It is important to note that we are predicting the extent to which PA contributes to the decline in BG levels. Only intervals showing a BG decrease were retained, as the initial focus of ACTIVE-GLU was to quantify PA-induced hypoglycaemic risk, the most clinically relevant and characterised direction of change in T1DM. This selection does not imply that PA universally lowers glucose; instead, it isolates the subset where a physiologically meaningful decline was empirically observed. We used the *filtered merged data*, focusing on instances in which BG levels declined in the next time interval. This allows us to assess the risk of hypoglycaemia. The resulting dataset, stored in the variable *actgluco*, contains both PA with four levels and BG data, and is now ready for developing our prediction model. The prediction model will forecast BG 15 minutes after performing various intensities of PA Algorithm 5.

To focus this on hypoglycaemia-related prediction, only intervals showing a BG decline (Δ*BG*(*t*) > 0) were used for model training. At the same time, the evaluation included all subsequent predictions, so instances in which BG later rose were counted as prediction failures. This filtering was applied once on the complete dataset before constructing any rolling windows or next-day forecasts, using only information available up to that time, thereby maintaining causality and avoiding look-ahead bias. The resulting dataset, consisting of synchronised 15-minute PA and BG pairs, provided a consistent and interpretable foundation for modelling PA-induced glucose changes.

### STROBE Checklist

**Appendix S1.** The completed STROBE checklist for cohort studies is provided below.

Item	Recommendation	Page
1a	Study design indicated in title/abstract	1
1b	Informative abstract summary	1
2	Background / rationale	1–2
3	Objectives	2
4	Study design	3
5	Setting	3–4
6a	Participants eligibility	3
7	Variables definition	4–6
8	Data sources / measurement	3–6
9	Bias	4–5
10	Study size	3
11	Quantitative variables	4–5
12	Statistical methods	6–7
13	Participants results	3, 9
14	Descriptive data	3
15	Outcome data	9–12
16	Main results	9–12
17	Other analyses	9–13
18	Key results	Discussion
19	Limitations	Discussion
20	Interpretation	Discussion
21	Generalisability	Discussion
22	Funding	Funding section
